# Linkage Isomerism Leading to Contrasting Carboboration Chemistry: Access to Three Constitutional Isomers of a Borylated Phosphaalkene

**DOI:** 10.1002/chem.202002226

**Published:** 2020-10-08

**Authors:** Daniel W. N. Wilson, Meera Mehta, Mauricio P. Franco, John E. McGrady, Jose M. Goicoechea

**Affiliations:** ^1^ Department of Chemistry University of Oxford Chemistry Research Laboratory 12 Mansfield Road Oxford OX1 3TA UK; ^2^ Department of Chemistry University of Manchester Oxford Road Manchester M13 9PL UK; ^3^ Instituto de Química University of São Paulo Av. Prof. Lineu Prestes, 748—Vila Universitaria São Paulo—SP 05508-000 Brazil

**Keywords:** boryl groups, carboboration, isomerism, phosphaalkenes, phosphaethynolates

## Abstract

We describe the reactivity of two linkage isomers of a boryl‐phosphaethynolate, [B]OCP and [B]PCO (where [B]=*N*,*N’*‐bis(2,6‐diisopropylphenyl)‐2,3‐dihydro‐1*H*‐1,3,2‐diazaboryl), towards tris‐ (pentafluorophenyl)borane (BCF). These reactions afforded three constitutional isomers all of which contain a phosphaalkene core. [B]OCP reacts with BCF through a 1,2 carboboration reaction to afford a novel phosphaalkene, *E*‐[B]O{(C_6_F_5_)_2_B}C=P(C_6_F_5_), which subsequently undergoes a rearrangement process involving migration of both the boryloxy and pentafluorophenyl substituents to afford *Z*‐{(C_6_F_5_)_2_B}(C_6_F_5_)C=PO[B]. By contrast, [B]PCO undergoes a 1,3‐carboboration process accompanied by migration of the *N*,*N’*‐bis(2,6‐diisopropylphenyl)‐2,3‐dihydro‐1*H*‐1,3,2‐diazaboryl to the carbon centre.

## Graphical abstract

**Isomerism**: The contrasting carboboration chemistry of two linkage isomers of a boryl‐functionalised phosphaethynolate towards tris(pentafluorophenyl)borane is explored leading to three constitutional isomers of a novel phosphaalkene.

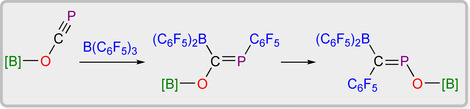

## Introduction

Despite its widespread use as the Lewis acidic component in frustrated Lewis pair systems, tris(pentafluorophenyl)borane [B(C_6_F_5_)_3_; BCF] is known to react in carboboration processes in which the B−C bond adds across unsaturated element−element bonds.^1^, ^2^, ^3^ While such transformations are often undesirable, they represent an attractive synthetic route to functionalized organoboron compounds, and as such, merit further investigation. The most well‐studied of known carboboration processes involving electrophilic boranes is the 1,1‐carboboration of alkynes,^4^ which has a precedent in the Wrackmeyer reaction.^5^ By contrast, 1,2‐carboboration reactions are rarer, and largely limited to metal‐catalysed processes.^6^ To date, only highly reactive borocations have been shown to give rise to metal‐free 1,2‐carboboration reactions with alkynes.^7^, ^8^ In the case of tris(pentafluorophenyl)borane, related reactions are only possible using more reactive substrates, such as allenyl ketones and isocyanates.^9^, ^10^, ^11^, ^12^ It is these latter studies, as well as related work exploring the hydroboration of phosphaalkynes,^13^, ^14^, ^15^ that prompted us to explore the reactivity of BCF towards two isomeric forms of a boryl‐phosphaethynolate, [B]OCP and [B]PCO (where [B]=*N*,*N’*‐bis(2,6‐diisopropylphenyl)‐2,3‐dihydro‐1*H*‐1,3,2‐diazaboryl).^16^, ^17^ Interestingly, these reactions give rise to three different borylated phosphaalkenes with the same molecular formula.

## Results and Discussion

**Scheme 1:**
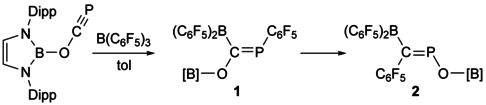
Reaction of [B]OCP towards B(C_6_F_5_)_3_ to afford **1** and its isomerization to **2**.

Addition of tris(pentafluorophenyl)borane (BCF) to a solution of [B]OCP resulted in an immediate colour change from yellow to dark red. The ^31^P{^1^H} and ^1^H NMR spectra of the reaction mixture are consistent with the formation of a single product with a ^31^P{^1^H} NMR resonance at 173.9 ppm (**1**, Scheme 1). The ^19^F{^1^H} NMR spectrum indicates the presence of two pentafluorophenyl environments in a 2:1 ratio. Analysis of single crystals obtained from this reaction revealed that 1,2‐carboboration of the C≡P bond of [B]OCP had taken place with formation of C−B and P−C bonds affording *E*‐[B]O{(C_6_F_5_)_2_B}C=P(C_6_F_5_) (**1**). This bond formation is inverse to that observed in the related hydroboration of phosphaalkynes using Piers’ borane, HB(C_6_F_5_)_2_, which forms C−H and P−B bonds due to the steric interaction of the C_6_F_5_ groups and the *tert*‐butyl of the phosphaalkyne.^15^ It is worth noting at this stage that previous reactions of phosphaethynolate salts with boranes were found to exclusively yield dimeric compounds with the boranes remaining intact.^18^

**Figure 1:**
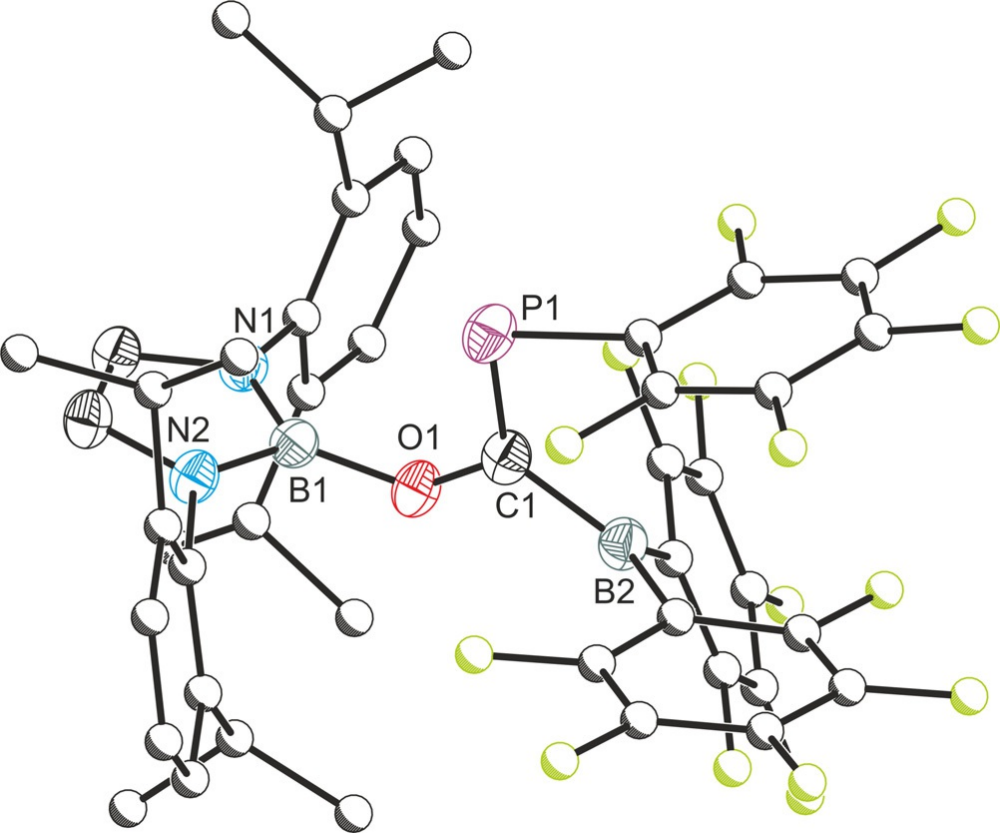
Molecular structure of **1**. Anisotropic displacement ellipsoids set at 50 % probability. Hydrogen atoms omitted for clarity. Atoms of the Dipp and C_6_F_5_ moieties pictured as spheres of arbitrary radius. Selected interatomic distances [Å] and angles [°]: B1−O1 1.404(2); O1−C1 1.356(2), C1−P1 1.701(2), C1−B2 1.577(3); B1‐O1‐C1 126.40(14); O1‐C1‐P1 121.24(13), O1‐C1‐B2 109.92(15), P1‐C1‐B2 128.69(14).

The single‐crystal X‐ray structure of **1** (Figure 1) revealed a *cis*‐ arrangement of the C_6_F_5_ and B(C_6_F_5_)_2_ groups and a C=P bond distance (1.701(2) Å) with significant double bond character (typically in the region of 1.69 Å).^19^ This distance is comparable to related boryl‐functionalized phosphaalkyes such as *Z*‐(HO)[B]C=PMes (1.699(2) Å),^16^ previously reported by our research group. The C−O bond, 1.356(2) Å, is notably longer than that of the [B]OCP precursor (1.269(2) Å) due to the loss of π‐conjugation between the phosphaethynolate and the *N*,*N’*‐bis(2,6‐diisopropylphenyl)‐2,3‐dihydro‐1*H*‐1,3,2‐diazaboryl moiety.

Given the inverse polarization of the C≡P bonds in phosphaalkynes relative to nitriles, that is, ^δ−^C≡P^δ+^ versus ^δ+^C≡N^δ−^, the stereo‐ and regio‐selectivity of this transformation is consistent with an interaction of the boron atom of BCF with the carbon centre of the boryloxy‐functionalized phosphaalkyne, [B]OCP, hence the final *cis*‐configuration of the C_6_F_5_ and B(C_6_F_5_)_2_ groups. Related 1,2‐carboboration reactions of phosphaalkynes have been previously reported by Martin and co‐workers for boron‐containing heterocycles such as 9‐borafluorene,^20^ whereas more complex rearrangements were observed for pentaarylboroles.^21^

**Figure 2:**
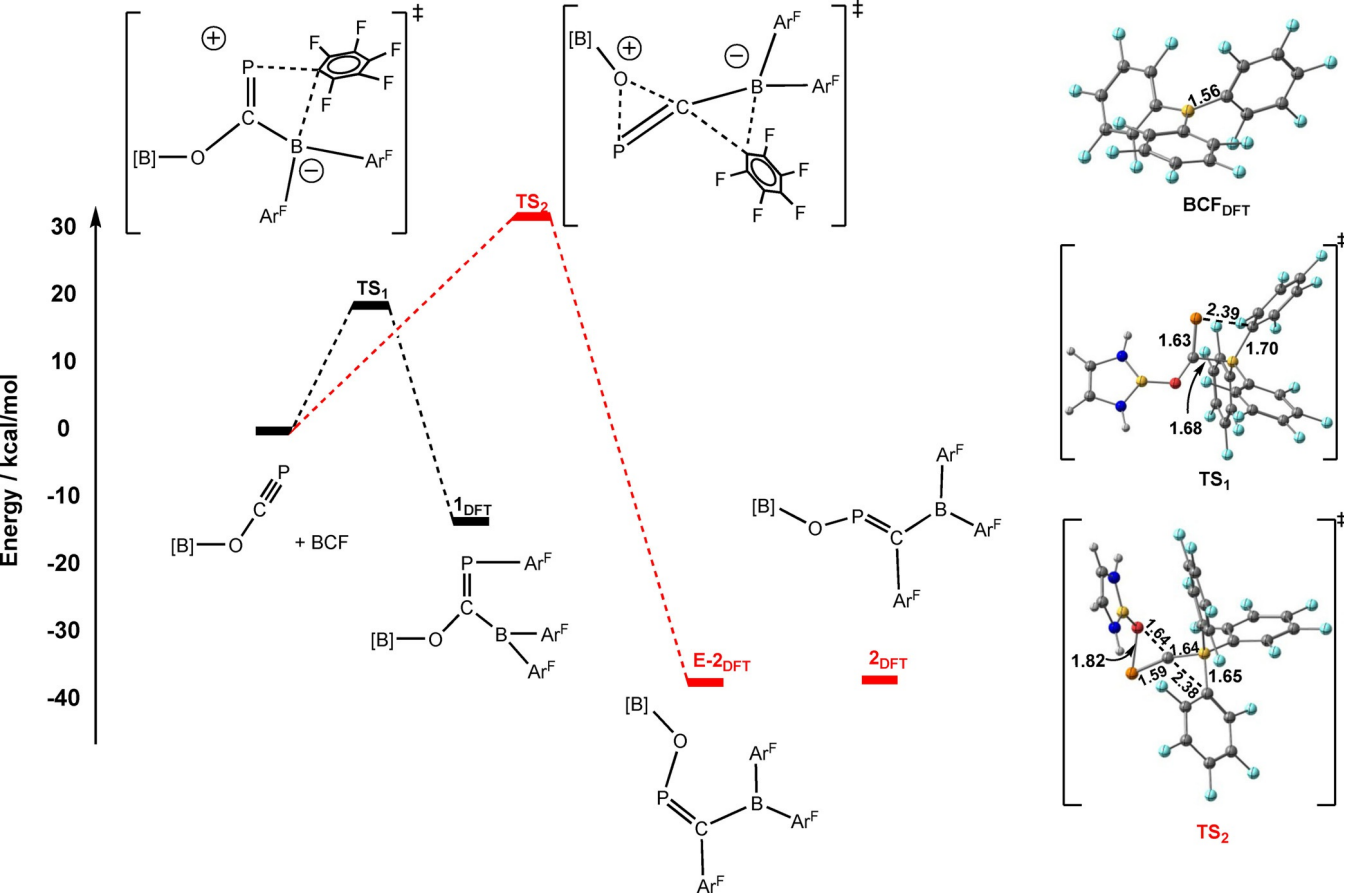
Relative energies of reactants, **1_DFT_**, **2_DFT_** and **E‐2_DFT_** and the proposed pathway for the formation of **1_DFT_** (black). The transition state **TS_2_** connects reactants to the *E*‐ isomer of **2**, but the overall barrier (red pathway) is too high to be consistent with a rapid reaction at room temperature.

In order to shed further light on the mechanism that leads to **1** we have performed a series of calculations using DFT. In all cases the 2,6‐diisopropylphenyl (Dipp) groups on the *N*,*N’*‐bis(2,6‐diisopropylphenyl)‐2,3‐dihydro‐1*H*‐1,3,2‐diazaboryl units are replaced by hydrogen for computational expedience. The computed bond lengths in the reactants and in the 1,2 carboboration product, **1_DFT_**, are very similar to those observed in the X‐ray diffraction experiments. The 1,2‐carboboration pathway proceeds through a transition state, **TS_1_**, which lies 18.8 kcal mol^−1^ above the reactants, consistent with a rapid reaction at room temperature (Figure 2). Ingleson and co‐workers reported a very similar barrier for the 1,2‐carboboration of 2‐butyne with a borenium cation.^7^ At the transition state, the BCF unit is tightly bonded to the carbon centre of the OCP ligand (B−C=1.68 Å vs. 1.57 Å in the product) and the P≡C triple bond is elongated by 0.08 Å, consistent with a phosphinidene‐like structure. In contrast, the new P−C_aryl_ bond is only at an early stage of formation (2.39 Å vs. a final value of 1.85 Å), suggesting that the barrier to the 1,2 reaction is associated primarily with the transfer of electron density from the P≡C triple bond to the boron centre of BCF. It is worth mentioning at this point that, in contrast to our observations, Longobardi et al. have shown previously that BCF is unreactive towards the phospha‐alkyne *t*BuCP.^15^ A comparison of the frontier orbitals of *t*BuCP and [B]OCP shows that the HOMO is C−P π‐based in both cases (Supporting Information, Figure S20), but the electron donating effect of the boryloxy substituent results in a ≈1.4 eV destabilisation (−6.45 eV in *t*BuCP vs. −5.03 eV in [B]OCP). The greater nucleophilicity of [B]OCP is clearly important in stabilizing the dominant charge transfer pathway leading to **TS_1_**.

**Figure 3:**
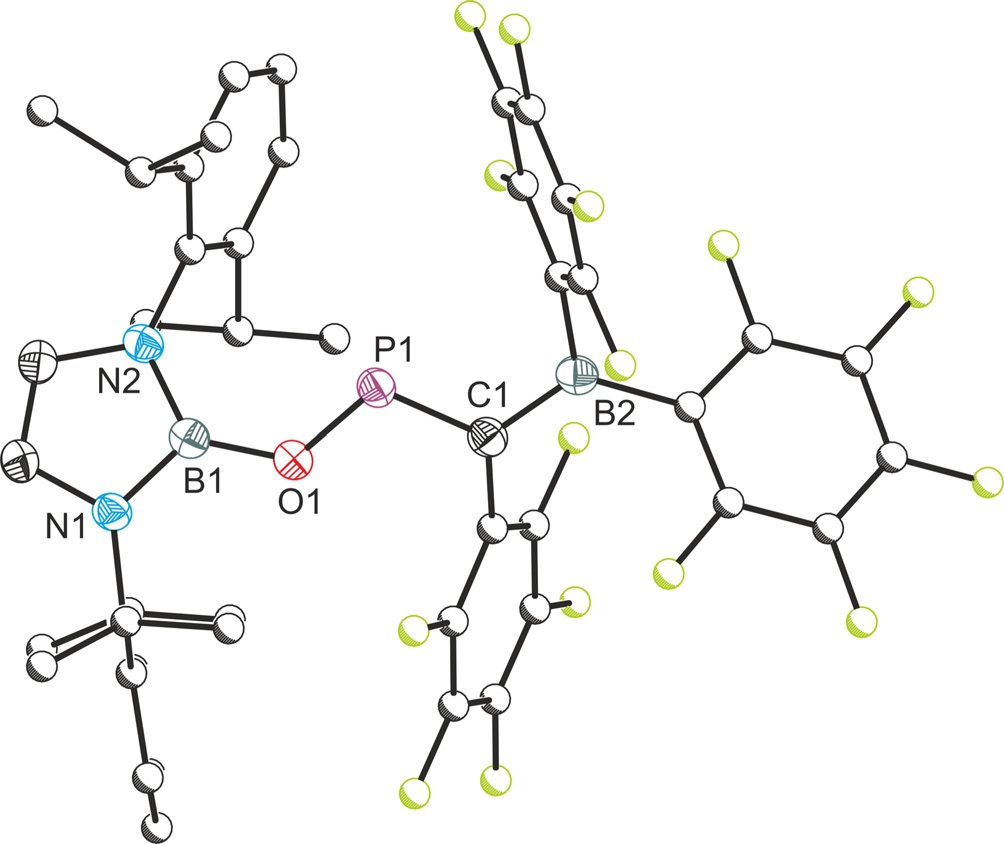
Molecular structure of **2**. Anisotropic displacement ellipsoids set at 50 % probability. Hydrogen atoms omitted for clarity. Atoms of the Dipp and C_6_F_5_ moieties pictured as spheres of arbitrary radius. Selected interatomic distances [Å] and angles [°]: B1−O1 1.398(2); O1−P1 1.593(2), P1−C1 1.684(2), C1−B2 1.512(2); B1‐O1‐P1 125.35(11); O1‐P1‐C1 104.06(7), P1‐C1‐B2 112.07(12).

Interestingly, **1** underwent a rearrangement in solution over the course of several hours to *Z*‐{(C_6_F_5_)_2_B}(C_6_F_5_)C=PO[B], **2**. The ^31^P{^1^H} NMR spectrum of **2** reveals a singlet at 401.9 ppm which is significantly shielded relative to **1** (173.9 ppm). As with **1**, two distinct groups of resonances (in a 2:1 ratio) are observed in the ^19^F{^1^H} NMR spectrum corresponding to the pentafluorophenyl functionalities. Analysis of the crystal structure of **2** (Figure 3) reveals a short C=P double bond (1.684(2) Å) which is comparable to that of **1** (1.701(2) Å). The B−O and O−P bonds, 1.398(2) and 1.593(2) Å, respectively, are shorter than conventional single bonds implying a significant degree of electron delocalization along the C‐P‐O‐B core, which may explain the high frequency chemical shift observed for this compound. DFT calculations reveal that **2** is the thermodynamic product of the reaction lying 28 kcal mol^−1^ lower in energy than its isomer **1**. Compound **2** shows no FLP‐type reactivity towards gases (H_2_, CO, CO_2_, CS_2_, N_2_O).

**Scheme 2:**
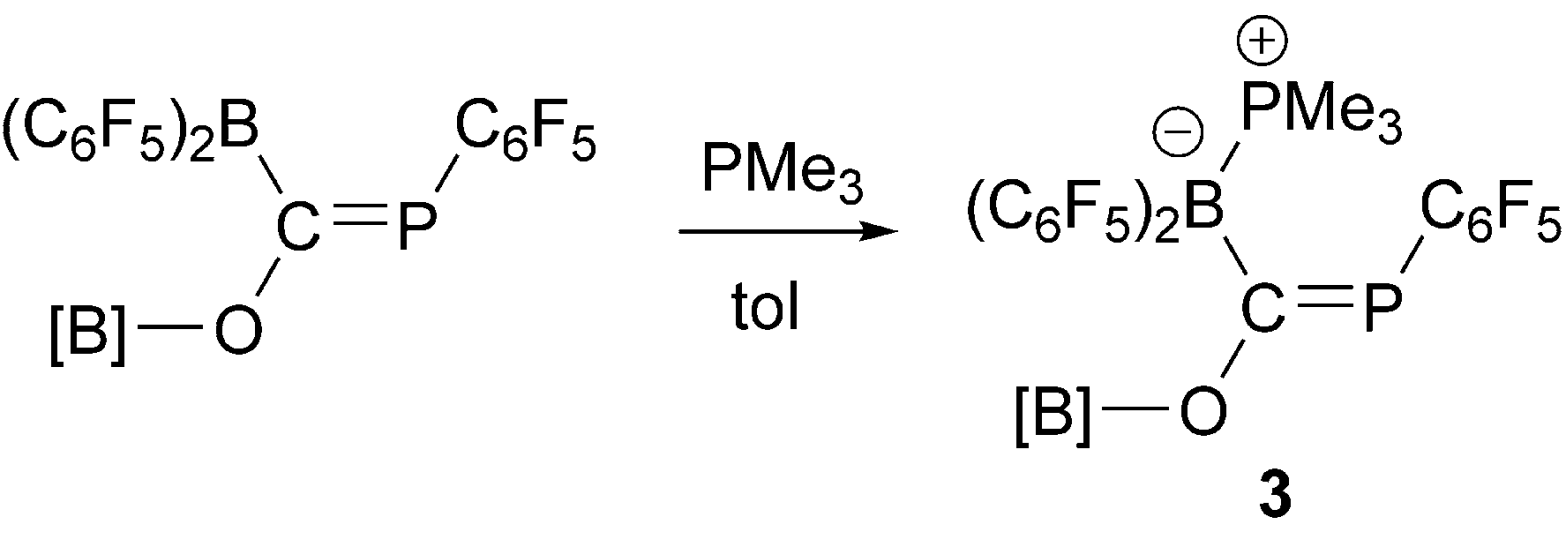
Reaction of **1** with PMe_3_ to afford **3**.

It is potentially significant that the isomerization of **1** to **2** can be prevented by addition of a Lewis base. This quenches the acidity of the B(C_6_F_5_)_2_ functionality, indicating that the empty p‐orbital on the boryl centre plays a key role in the isomerization. Addition of one equivalent of PMe_3_ to a solution of **1** affords the acid‐base adduct **3** (Scheme 2) in quantitative yield. In contrast to **1**, this species was found to be indefinitely stable in solution. Compound **3** exhibits two resonances in the ^31^P{^1^H} NMR spectrum at 145.4 and −10.7 ppm corresponding to the phosphorus atoms of the phosphalkene and the coordinated trimethylphosphine, respectively. The former is moderately shifted relative to that of **1** (173.9 ppm). All other spectroscopic properties are largely in line with those observed for the 1,2‐carboboration product. Single crystals suitable for X‐ray diffraction of **3** were grown from a concentrated hexane solution stored at −35 °C for 5 days (see Supporting Information for details). The B−C bond of **3** is notably elongated relative to **1** (1.638(2) vs. 1.577(3) Å) indicating a lack of π‐orbital overlap between the B(C_6_F_5_)_2_ group and the carbon centre.

Our calculations indicate that the optimised structure of **2_DFT_** is 23 kcal mol^−1^ more stable than **1_DFT_**, the product of the 1,2‐carboboration, and so the former is clearly the thermodynamic product of the reaction. Despite multiple efforts, we have, however, been unable to locate a transition state that connects **1_DFT_** and **2_DFT_** directly. Following Ingleson's work which reported a 1,1‐carboboration pathway, we have located a similar transition state, **TS2**, where the [B]O group migrates from the carbon to the phosphorus. However, **TS2** connects the reactants to the *E* isomer of **2**, ***E*** **‐2_DFT_**, rather to than **2** itself, and with a rather high barrier of 31 kcal mol^−1^. Given that the total barrier would be augmented by the energy required to reverse the formation of **1**, (Δ*E*=+16 kcal mol^−1^), it seems unlikely that it represents the true pathway that connects **1** and **2**. In the absence of a viable unimolecular rearrangement pathway, we speculate that the rearrangement may occur via a dimeric pathway, with the Lewis acidity of the B(C_6_F_5_)_2_ functionality playing a role in stabilizing the dimer. Efforts to identify such a pathway are ongoing.

**Scheme 3:**
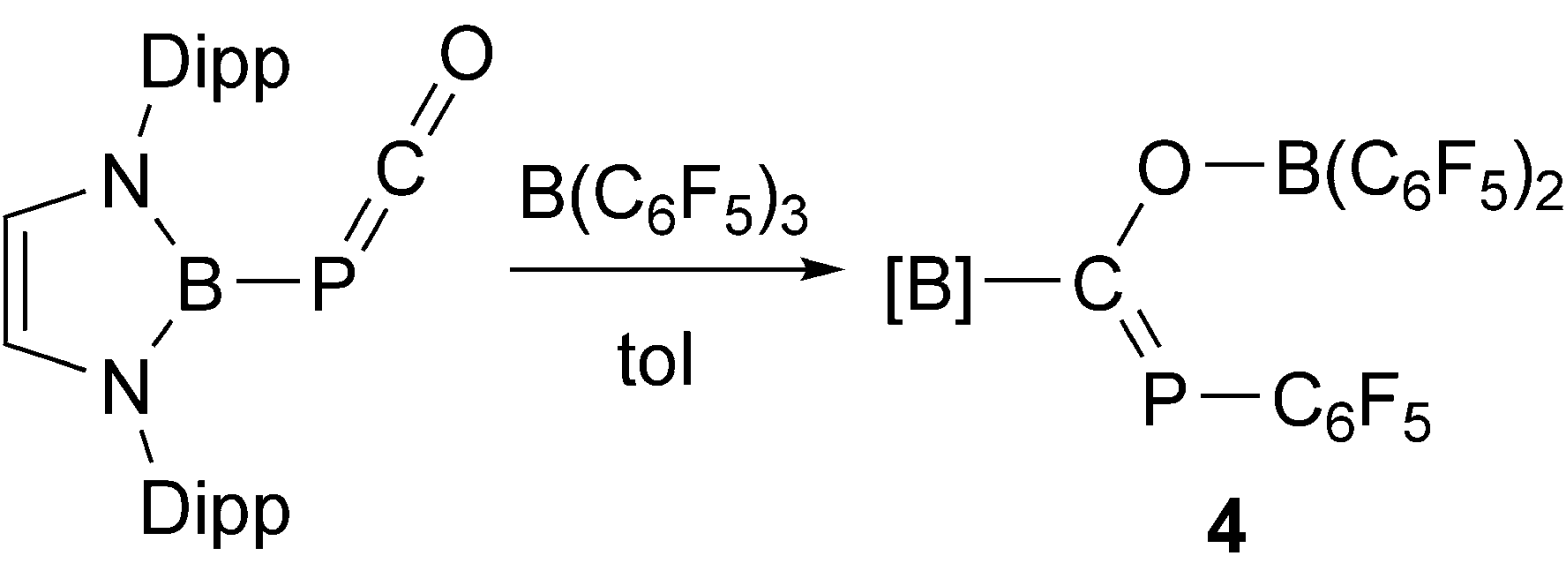
Reaction of [B]PCO towards B(C_6_F_5_)_3_ to afford **4**.

**Figure 4:**
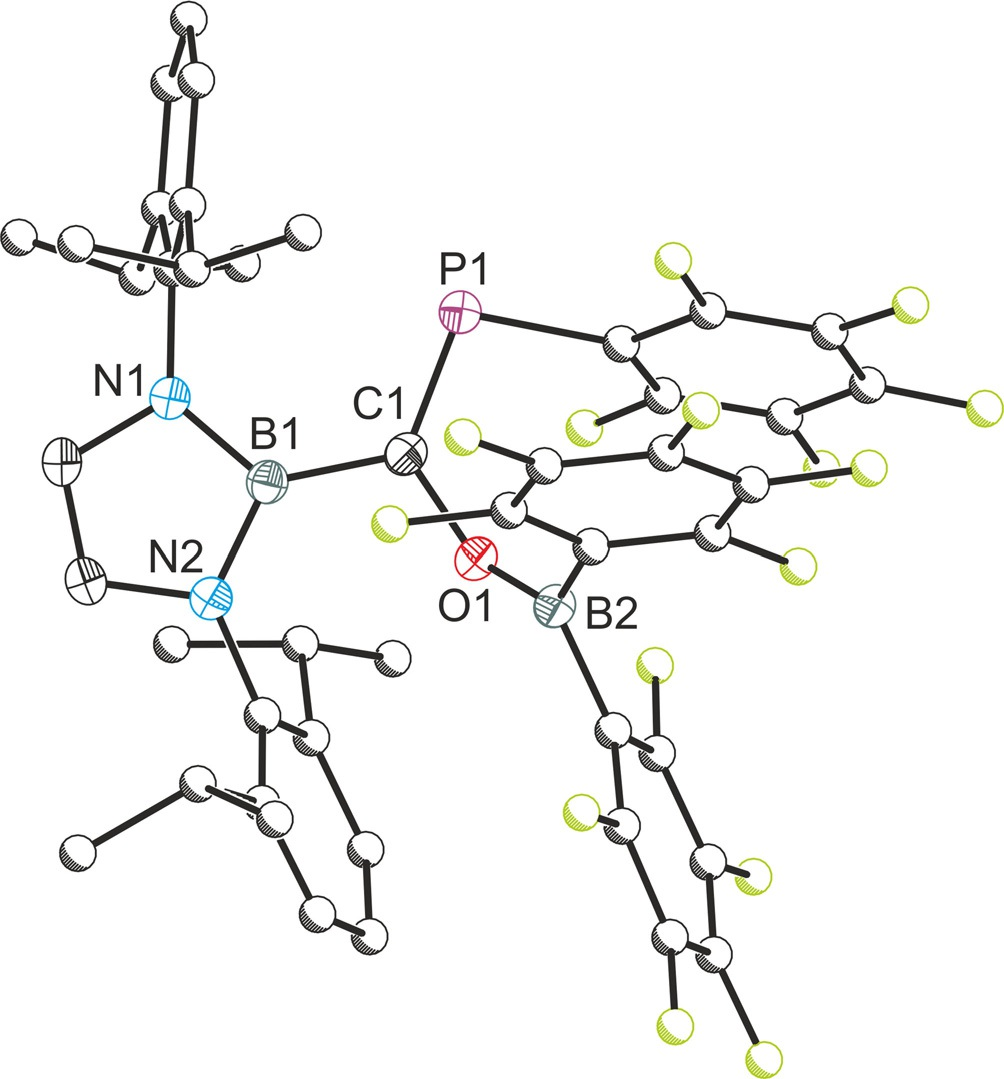
Molecular structure of **4**. Anisotropic displacement ellipsoids set at 50 % probability. Hydrogen atoms are been omitted for clarity. Atoms of the Dipp and C_6_F_5_ moieties are pictured as spheres of arbitrary radius. Selected interatomic distances [Å] and angles [°]: B1−C1 1.566(2); C1−P1 1.692(2), C1−O1 1.393(2), O1−B2 1.341(2); B1‐C1‐P1 122.52(9); B1‐C1‐O1 112.59(10), P1‐C1‐O1 124.31(9); C1‐O1‐B2 130.72(10).

Addition of one equivalent of BCF to the linkage isomer of [B]OCP, [B]PCO, leads to quantitative formation of a new product, **4**, with a singlet in its ^31^P{^1^H} NMR spectrum at 142.2 ppm (Scheme 3). Crystals were obtained from a cooled hexane solution and the structure revealed a carboboration reaction in which the boryl has migrated from the phosphorus to the phosphaketenyl carbon accompanied by O−B and P−C bond formation (Figure 4). It is worth noting that we have previously observed boryl group migration in reactions of both [B]OCP with nucleophiles,^16^, ^22^ however that this is the first instance of such migration reactions occurring with its isomeric form [B]PCO. The carboboration of isocyanates, valence isoelectronic analogues of phosphaketenes, was recently found to give rise to 1,2‐carboborated products across the C=O bond, which insert a second equivalent of isocyanate to yield six‐membered heterocycles.^10^, ^11^ In contrast, related isoelectronic compounds, such as hydrazoic acid and organic azides react with BCF via a 1,1‐carboboration accompanied by loss of dinitrogen to afford aminoboranes.^23^

The P−C bond length is again consistent with significant π‐character (1.692(2) Å).^24^ Of note is the shorter than usual B−O bond which, at 1.341(2) Å, is closer to what is expected for a double bond (1.45/1.35 Å for single and double bonds, respectively).^19^ This contraction is consistent with significant donation from the oxygen lone pair into the empty p orbital of the boryl group, which is now devoid of competing π‐donor substituents.

## Conclusions

We have shown that tris(pentafluorophenyl)borane reacts readily with two isomers of a boryl‐phosphaethynolate, [B]OCP and [B]PCO (where [B]=*N*,*N’*‐bis(2,6‐diisopropylphenyl)‐2,3‐dihydro‐1*H*‐1,3,2‐diazaboryl). In the case of the former isomer, which can be thought of as a boryloxy‐functionalized phosphalkyne, a concerted 1,2‐carboboration reaction is observed initially, however the product ultimately rearranges to a more thermodynamically stable constitutional isomer. By contrast the linkage isomer [B]PCO, a boryl‐functionalized phosphaketene, undergoes a formal 1,3‐carboboration in which O−B(C_6_F_5_)_2_ and P−C_6_F_5_ bonds are formed accompanied by migration of the boryl functionality from phosphorus to carbon. The three resulting products from these reactions are all isomers of the same borylated phosphaalkenes and allow for a mechanistic probe of the mechanism of carboboration.

## Experimental Section

All reactions and product manipulations were carried out under an inert atmosphere of argon or dinitrogen using standard Schlenk‐line or glovebox techniques (MBraun UNIlab glovebox maintained at <0.1 ppm H_2_O and <0.1 ppm O_2_). [B]OCP, [B]PCO and tris(pentafluorophenyl)borane were synthesized according to previously reported synthetic procedures.1b, 16, 17 Hexane (hex; Sigma Aldrich, HPLC grade), and toluene (Sigma Aldrich, HPLC grade) were purified using an MBraun SPS‐800 solvent system. C_6_D_6_ (Aldrich, 99.5 %) was degassed prior to use. All dry solvents were stored under argon in gas‐tight ampoules. All solvents were stored over 3 Å molecular sieves.

NMR spectra were acquired on a Bruker AVIII 500 MHz NMR spectrometer (^1^H 500 MHz, ^13^C 126 MHz) and Bruker AVIII 400 MHz NMR spectrometer (^1^H 400 MHz, ^31^P 162 MHz, ^11^B 128 MHz, ^19^F 376 MHz). ^1^H and ^13^C NMR spectra were referenced to the most downfield solvent resonance (^1^H NMR C_6_D_6_: *δ*=7.16 ppm; ^13^C NMR C_6_D_6_: *δ*=188.06 ppm). ^31^P, ^19^F and ^11^B spectra were externally referenced to an 85 % solution of H_3_PO_4_ in H_2_O, CFCl_3_ and BF_3_ **⋅**Et_2_O in C_6_D_6_ respectively. Elemental analyses were carried out by Elemental Microanalyses Ltd. (Devon, U.K.). Samples (approx. 5 mg) were submitted in sealed Pyrex ampoules. Full details of the computational methods can be found in the Supporting Information.

Synthesis of *E*‐[B]O{(C_6_F_5_)_2_B}C=P(C_6_F_5_) (**1**): Tris(pentaflourophenyl)borane (110 mg, 0.22 mmol) was added to a solution of [B]OCP (100 mg, 0.22 mmol) in toluene (3 mL). The solution immediately changed colour from pale yellow to red. Immediate removal of the solvent, followed by dissolution in hexane (2 mL) and cooling to −35 °C overnight yielded red crystals of **1** suitable for single‐crystal X‐ray diffraction. NMR of the crystals revealed a mixture of compounds **1** and **2**. Partial NMR data for **1** was obtained from the reaction mixture, with ≈10 % of [B]OCP present. Given the propensity for **1** to isomerize to **2** in solution, a compositionally pure sample of this compound could not be isolated. ^1^H NMR (400 MHz, C_6_D_6_): δ (ppm) 7.33–7.26 (m, 2 H; *para*‐ArH), 7.15 (m, 4 H; *meta*‐ArH), 6.17 (s, 2 H; {(NC*H*)_2_}), 3.33 (sept, ^3^ *J*_H‐H_=6.5 Hz, 4 H; {C*H*(CH_3_)_2_}), 1.27 (d, ^3^ *J*_H‐H_=6.9 Hz, 12 H; {CH(C*H*_3_)_2_}), 1.19 (d, ^3^ *J*_H‐H_=6.8 Hz, 12 H; {CH(CH_3_)_2_}). ^11^B NMR (128 MHz, C_6_D_6_): δ (ppm) 21.40 (s, br). ^19^F NMR (376 MHz, C_6_D_6_): δ (ppm) −125.80 (d, ^3^ *J*_F‐F_=24.5 Hz, 2F; *ortho*‐P(C_6_F_5_)), −126.17 (d, ^3^ *J*_F‐F_=20.0 Hz, 4F; *ortho*‐B(C_6_F_5_)_2_), −144.39 (tt, ^3^ *J*_F‐F_=20.7 Hz, ^4^ *J*_F‐F_=5.8 Hz, 2F; *para*‐B(C_6_F_5_)_2_), −150.60 (t, ^3^ *J*_F‐F_=20.5 Hz, 1F; *para*‐P(C_6_F_5_)), −160.26 to −160.70 (m, 6F; *meta*‐B(C_6_F_5_)_2_ and *meta*‐P(C_6_F_5_)). ^31^P NMR (162 MHz, C_6_D_6_): δ (ppm) 173.9.

Synthesis of *Z*‐{(C_6_F_5_)_2_B}(C_6_F_5_)C=PO[B] (**2**): Tris(pentaflourophenyl)borane (110 mg, 0.22 mmol) was added to a solution of [B]OCP (100 mg, 0.22 mmol) in toluene (3 mL). The solution immediately changed colour from pale yellow to red. The solution was stirred for 24 hours at room temperature. The solvent was removed and the resulting red oily solid was taken into hexane (5 mL) and filtered to afford an orange solution which was concentrated and cooled to −35 °C. After 7 days yellow crystals of **2** had formed (148 mg, 70.5 % yield). Crystallization from toluene afforded a different solvate, **2⋅**0.5 tol. CHN Anal. Calcd. for C_45_H_36_B_2_F_15_N_2_OP: C, 56.40 %; H, 3.79 %; N, 2.92 %; Found: C, 56.32 %; H, 3.84 %; N, 3.16 %. ^1^H NMR (400 MHz, C_6_D_6_): δ (ppm) 7.22 (t, ^3^ *J*_H‐H_=7.7 Hz, 2 H; *para*‐ArH), 7.00 (d, ^3^ *J*_H‐H_=7.8 Hz, 4 H; *meta*‐ArH), 5.83 (s, 2 H; {(NC*H*)_2_}), 2.93 (sept, ^3^ *J*_H‐H_=6.9 Hz, 4 H; {C*H*(CH_3_)_2_}), 1.07 (d, ^3^ *J*_H‐H_=6.9 Hz, 4.4 Hz, 12 H; {CH(C*H*_3_)_2_}), 1.05 (d, ^3^ *J*_H‐H_=6.9 Hz, 4.4 Hz, 12 H; {CH(C*H*_3_)_2_}). ^13^C NMR (126 MHz, C_6_D_6_): δ (ppm) 155.69 (d, ^1^ *J*_C‐P_=68.2 Hz; *C*P), 147.20 (C_6_F_5_), 145.87 (ArC), 145.25 (C_6_F_5_), 142.47 (C_6_F_5_), 140.52 (C_6_F_5_), 138.24 (C_6_F_5_), 136.23 (C_6_F_5_), 135.38 (ArC), 128.24 (ArC), 123.52 (ArC), 117.67 (C_6_F_5_), 117.07 ({(N*C*H)_2_}), 111.93 (C_6_F_5_), 28.43 ({*C*H(CH_3_)_2_}), 23.91 ({CH(*C*H_3_)_2_}), 23.01 ({CH(*C*H_3_)_2_}). ^11^B NMR (128 MHz, C_6_D_6_): δ (ppm) 21.10 (br, s). ^19^F NMR (376 MHz, C_6_D_6_): δ (ppm) −129.69 (br s; *ortho*‐P(C_6_F_5_)), −130.81 (br s; *ortho*‐P(C_6_F_5_)), −138.52 (d, ^3^ *J*_F‐F_=21.0 Hz; *ortho*‐B(C_6_F_5_)_2_), −146.81 (br s; *para*‐P(C_6_F_5_)), −150.99 (br s), −155.52 (t, ^3^ *J*_F‐F_=21.5 Hz; *para*‐B(C_6_F_5_)_2_), −159.43 to −162.64 (m; *meta*‐B(C_6_F_5_)_2_ and *meta*‐(C_6_F_5_)). [Note: Integrations omitted due to significant broadening in the spectrum.] ^31^P NMR (162 MHz, C_6_D_6_): δ (ppm) 401.9 (s).

Synthesis of *E*‐[B]O{(C_6_F_5_)_2_B(PMe_3_)}C=P(C_6_F_5_) (**3**): Tris(pentaflourophenyl)borane (28 mg, 0.06 mmol) was added to a solution of [B]OCP (25 mg, 0.06 mmol) in toluene (1 mL). The solution immediately changed colour from pale yellow to red. Addition of trimethylphosphine (1 m in toluene, 0.1 mL, 0.10 mmol) resulted in the immediate colour change from red to pale yellow. Excess trimethylphosphine and toluene were removed under reduced pressure and the resulting light‐yellow powder extracted into hexane. The eluent was filtered and cooled to −35 °C overnight to yield pale yellow crystals of **3** (29 mg, 54.7 %). CHN Anal. Calcd. for C_48_H_45_B_2_F_15_N_2_OP_2_: C, 55.73 %; H, 4.38 %; N, 2.71 %. Found: C, 56.53 %; H, 4.73 %; N, 2.71 %. ^1^H NMR (400 MHz, C_6_D_6_): δ (ppm) 7.37–7.28 (m, 2 H; *para*‐ArH), 7.18 (s, 4 H; *meta*‐ArH), 6.11 (s, 2 H; {(NC*H*)_2_}), 3.49 (sept, ^3^ *J*_H‐H_=6.4 Hz, 4 H; {C*H*(CH_3_)_2_}), 1.25 (d, ^3^ *J*_H‐H_=6.8 Hz, 12 H; {CH(C*H*_3_)_2_}), 1.19 (d, ^3^ *J*_H‐H_=6.8 Hz, 12 H; {CH(C*H*_3_)_2_}), 0.41 (d, ^2^ *J*_P‐H_=11.2 Hz, 9 H; PMe_3_). ^13^C{^1^H} NMR (126 MHz, C_6_D_6_): δ (ppm) 149.50 (C_6_F_5_), 147.59 (C_6_F_5_), 146.57 (ArC), 144.66 (C_6_F_5_), 141.17 (C_6_F_5_), 139.15 (C_6_F_5_), 138.37 (ArC), 136.26 (C_6_F_5_), 124.17 (ArC), 119.70 ({(N*C*H)_2_}), 28.94 ({*C*H(CH_3_)_2_}), 26.37 ({CH(*C*H_3_)_2_}), 23.16 ({CH(*C*H_3_)_2_}), 10.95 (d, ^1^ *J*_C‐P_=38.2 Hz, P(*C*H_3_)_3_). ^11^B NMR (128 MHz, C_6_D_6_): δ (ppm) 23.23 (br s), −12.99 (s, {(PMe_3_)B(C_6_F_5_)_2_}). ^19^F NMR (376 MHz, C_6_D_6_): δ (ppm) −152.62 (t, ^3^ *J*_F‐F_=20.7 Hz; *ortho*‐(C_6_F_5_)), −155.96 (t, ^3^ *J*_F‐F_=20.2 Hz, *para*‐(C_6_F_5_)), −161.60 (br s; *meta*‐(C_6_F_5_)). [Note: Integrations omitted due to significant broadening in the spectrum.] ^31^P NMR (162 MHz, C_6_D_6_): δ (ppm) 145.4 (d, ^3^ *J*_P‐P_=14.7 Hz; P=C), −10.7 (br s; PMe_3_).

Synthesis of *Z*‐(C_6_F_5_)P=C[B]{OB(C_6_F_5_)_2_} (**4**): Tris(pentaflourophenyl)borane (110 mg, 0.22 mmol) was added to a solution of [B]PCO (100 mg, 0.22 mmol) in toluene (3 mL). The solution darkened in colour. Removal of the solvent under a dynamic vacuum yielded a yellow powder. The solid was dissolved in hexane (5 mL), filtered and concentrated. Cooling the orange solution to −35 °C for 5 days resulted in yellow crystals of **4** (142 mg, 67.6 % yield). CHN Anal. Calcd. for C_45_H_36_B_2_F_15_N_2_OP: C, 56.40 %; H, 3.79 %; N, 2.92 %; Found: C, 57.06 %; H, 3.71 %; N, 3.80 %. ^1^H NMR (500 MHz, C_6_D_6_): δ (ppm) 6.99–6.79 (m, 6 H; ArH), 6.19 (s, 2 H; {(NC*H*)_2_}), 3.20 (sept, ^3^ *J*_H‐H_=6.6 Hz, 4 H; {C*H*(CH_3_)_2_}), 1.33 (d, ^3^ *J*_H‐H_=6.8 Hz, 12 H; {CH(C*H*_3_)_2_}), 1.12 (d, ^3^ *J*_H‐H_=6.8 Hz, 12 H; {CH(C*H*_3_)_2_}). ^13^C{^1^H} NMR (126 MHz, C_6_D_6_): δ (ppm) 202.32 (d, poorly resolved, ^1^ *J*_C‐P_=76 Hz, [B]*C*), 148.91 (C_6_F_5_), 146.95 (C_6_F_5_), 146.26 (C_6_F_5_), 145.36 (ArC), 144.41 (C_6_F_5_), 142.97 (C_6_F_5_), 142.46 (C_6_F_5_), 140.93 (C_6_F_5_), 138.55 (ArC), 138.30(C_6_F_5_), 136.31 (C_6_F_5_), 123.56 (ArC), 120.96 (ArC), 109.24 (C_6_F_5_), 108.72 (C_6_F_5_), 107.46 (C_6_F_5_), 28.36 ({*C*H(CH_3_)_2_}), 25.76 ({CH(*C*H_3_)_2_}), 22.35 ({CH(*C*H_3_)_2_}). ^19^F NMR (376 MHz, C_6_D_6_): δ (ppm) −128.46 (br s, weak), −147.23 (br s), −151.05 (t, ^3^ *J*_F‐F_=20.7 Hz), −160.78 (br s), −161.19 (br s). [Note: Integrations and assignment omitted due to significant broadening in the spectrum.] ^11^B NMR (128 MHz, C_6_D_6_): δ (ppm) 22.37 (br s). ^31^P NMR (162 MHz, C_6_D_6_): δ (ppm) 142.2.

**X‐ray diffraction**: Single‐crystal X‐ray diffraction data were collected using an Oxford Diffraction Supernova dual‐source diffractometer equipped with a 135 mm Atlas CCD area detector. Crystals were selected under Paratone‐N oil, mounted on micromount loops and quench‐cooled using an Oxford Cryosystems open flow N_2_ cooling device. Data were collected at 150 K using mirror monochromated Cu_Kα_ radiation (*λ*=1.5418 Å) and processed using the CrysAlisPro package, including unit cell parameter refinement and inter‐frame scaling (which was carried out using SCALE3 ABSPACK within CrysAlisPro).^25^ Equivalent reflections were merged and diffraction patterns processed with the CrysAlisPro suite. Structures were subsequently solved using direct methods and refined on *F* ^2^ using the SHELXL package.^26^ Further details of the crystallographic analyses described in this article can be found in the Supporting Information.

Deposition Numbers 2001083 (**1**), 2001084 (**2**), 2001085 (**2**⋅tol), 2001086 (**3**), and 2001087 (**4**)  contain the supplementary crystallographic data for this paper. These data are provided free of charge by the joint Cambridge Crystallographic Data Centre and Fachinformationszentrum Karlsruhe Access Structures service www.ccdc.cam.ac.uk/structures.

## Conflict of interest

The authors declare no conflict of interest.

## Supporting information

As a service to our authors and readers, this journal provides supporting information supplied by the authors. Such materials are peer reviewed and may be re‐organized for online delivery, but are not copy‐edited or typeset. Technical support issues arising from supporting information (other than missing files) should be addressed to the authors.

Supplementary
