# Supplementary material for: Linkage Isomerism Leading to Contrasting Carboboration Chemistry: Access to Three Constitutional Isomers of a Borylated Phosphaalkene
Source: Chemistry. 2020 Oct 8;26(59):13462–7. doi: 10.1002/chem.202002226 (PMC7702093; doi:10.1002/chem.202002226)
Supplement: Supplementary file 1 — Supplementary [file CHEM-26-13462-s001.pdf]

Chemistry–A European Journal

Supporting Information

Linkage Isomerism Leading to Contrasting Carboboration Chemistry: Access to Three Constitutional Isomers of a Borylated Phosphaalkene

Daniel W. N. Wilson,[a] Meera Mehta,[b] Mauricio P. Franco,[c] John E. McGrady,[a] and Jose M. Goicoechea*[a]

Supporting Information

Linkage isomerism leading to contrasting carboboration chemistry: Access to three constitutional isomers of a borylated phosphaalkene.

Daniel W. N. Wilson, Meera Mehta, Mauricio P. Franco, John E. McGrady and Jose M. Goicoechea*

E-mail: jose.goicoechea@chem.ox.ac.uk

Contents

1. NMR data for compounds **1**–**4**…………………….. 2–12

2. Single crystal X-ray diffraction data………………. 13–15

3. Computational details……………………………… 16–23

4. References…………………………………………. 24

1. NMR data for compounds 1–4

1.1 NMR data for ([B]O){(C_6F_5)$_2$B}C=P(C_6F_5) (**1**)

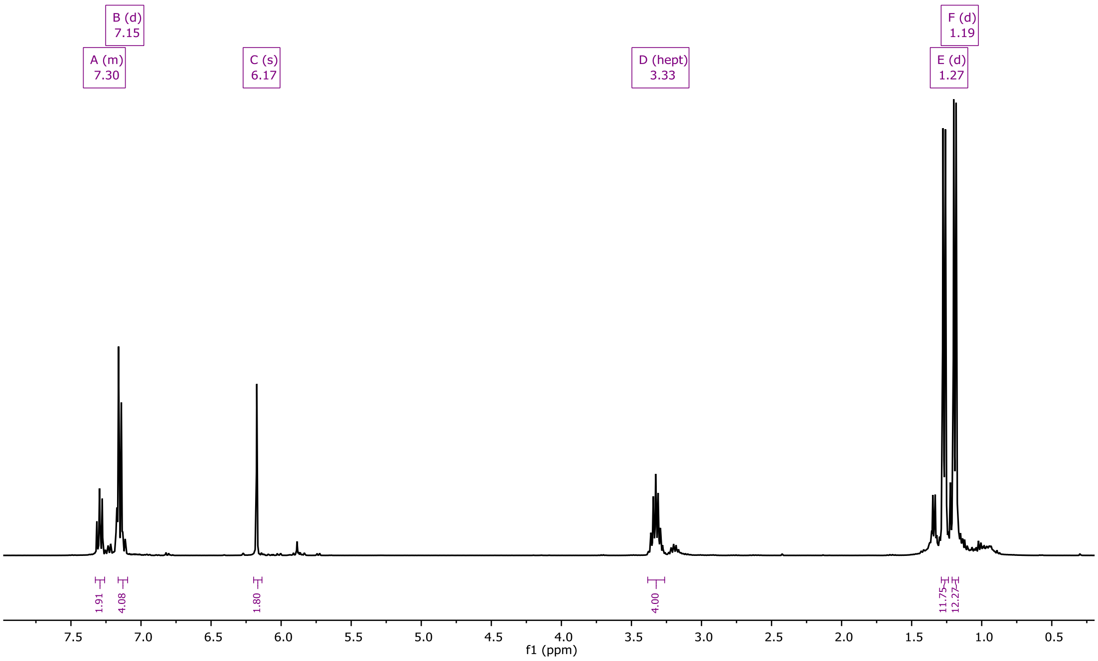

Figure S1. Room temperature ^{1}H NMR spectrum of **1** in C_6D_6.

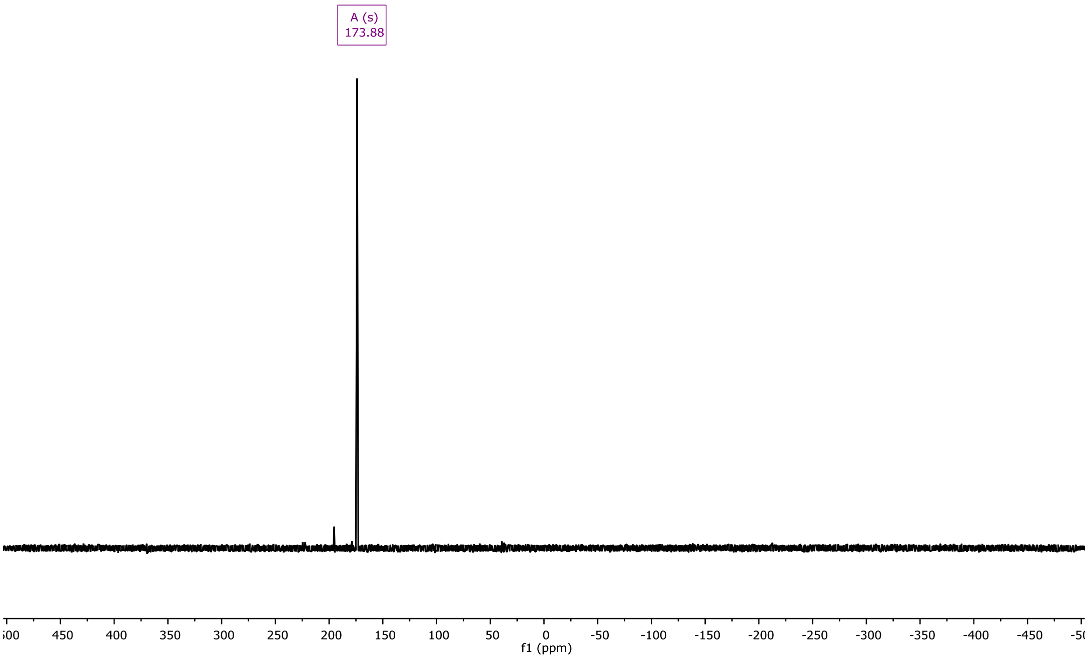

Figure S2. Room temperature ^{31}P NMR spectrum of **1** in C_6D_6.

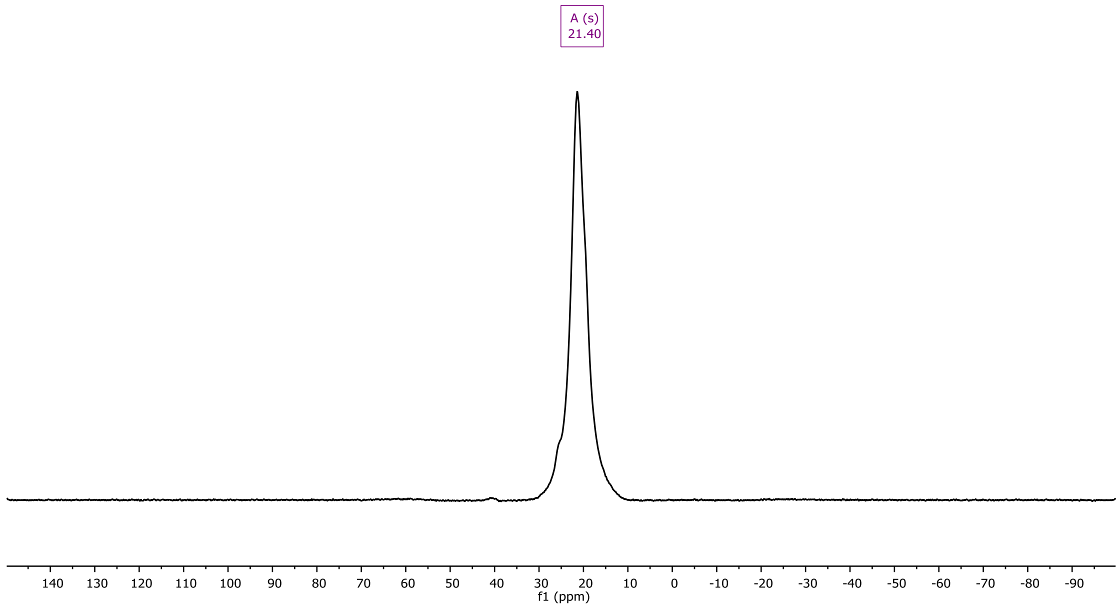

Figure S3. Room temperature ^{11}B NMR spectrum of **1** in C_6D_6.

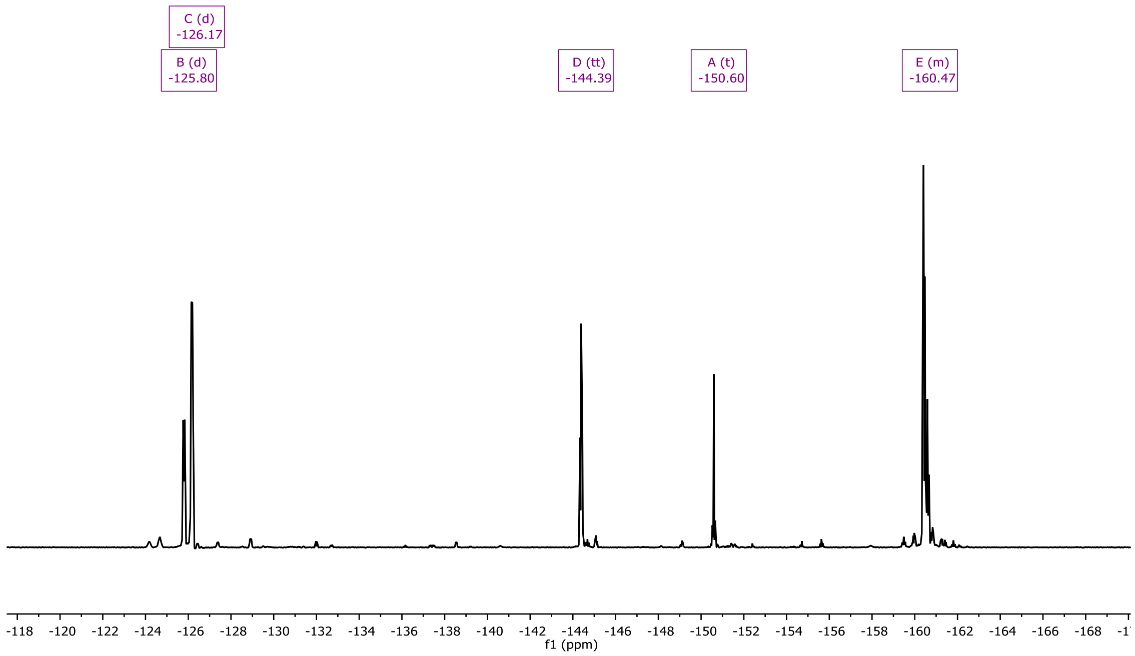

Figure S4. Room temperature ^{19}F NMR spectrum of **1** in C_6D_6.

1.2 NMR data for [B]OP=C(C_6H_5){B(C_6F_5)$_2$} (**2**)

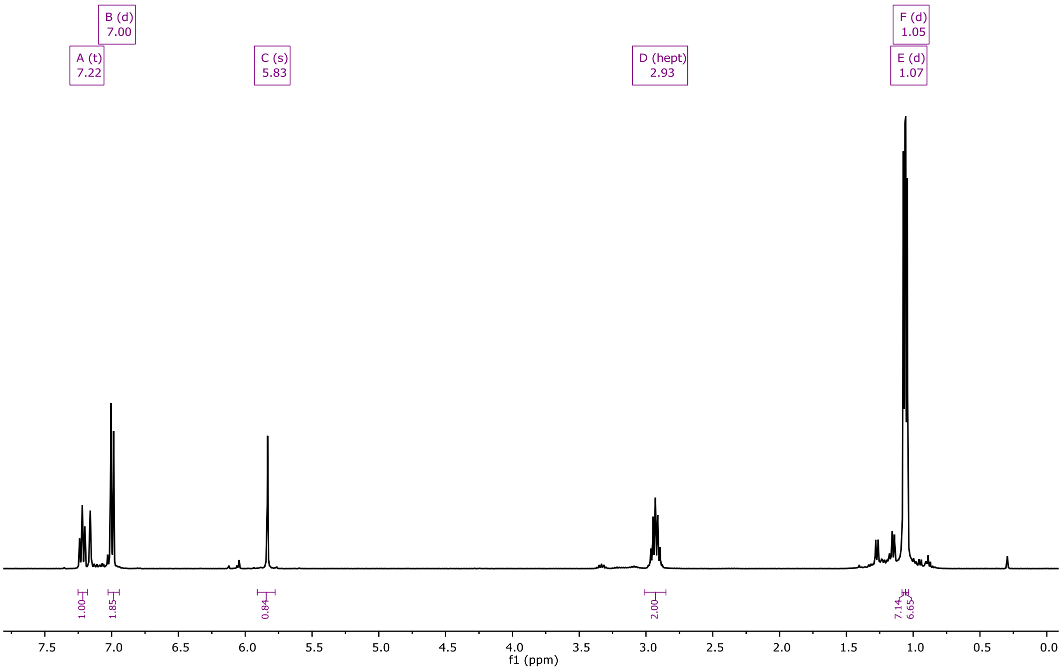

Figure S5. Room temperature ^{1}H NMR spectrum of **2** in C_6D_6.

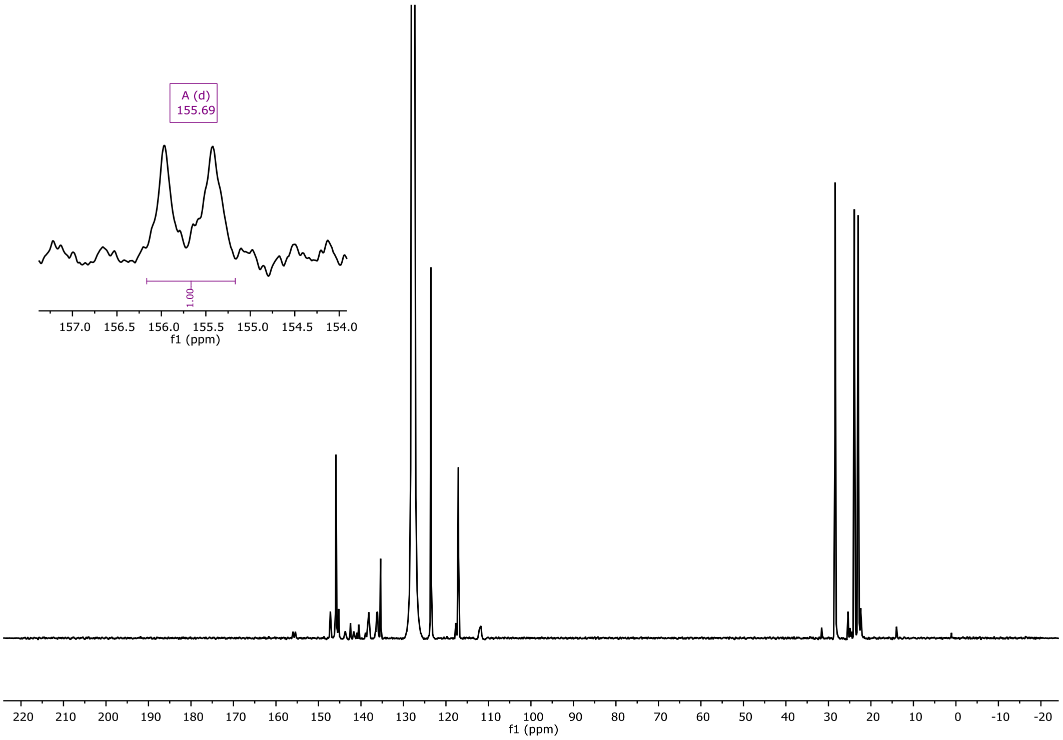

Figure S6. Room temperature ^{13}C{^{1}H} NMR spectrum of **2** in C_6D_6.

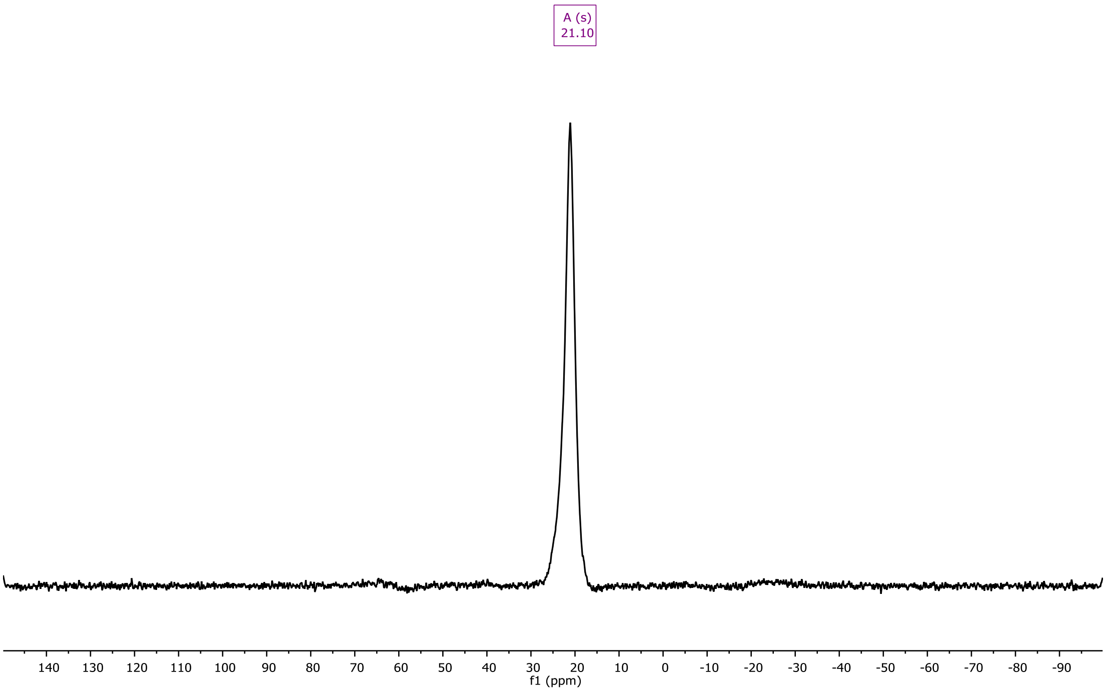

Figure S7. Room temperature ^{11}B NMR spectrum of **2** in C_6D_6.

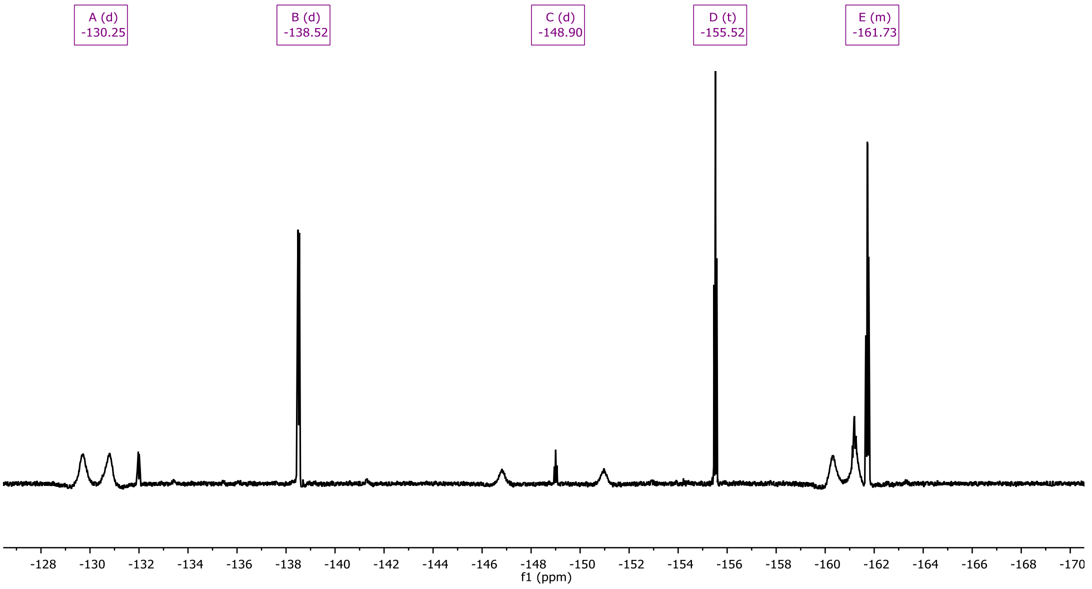

Figure S8. Room temperature ^{19}F NMR spectrum of **2** in C_6D_6.

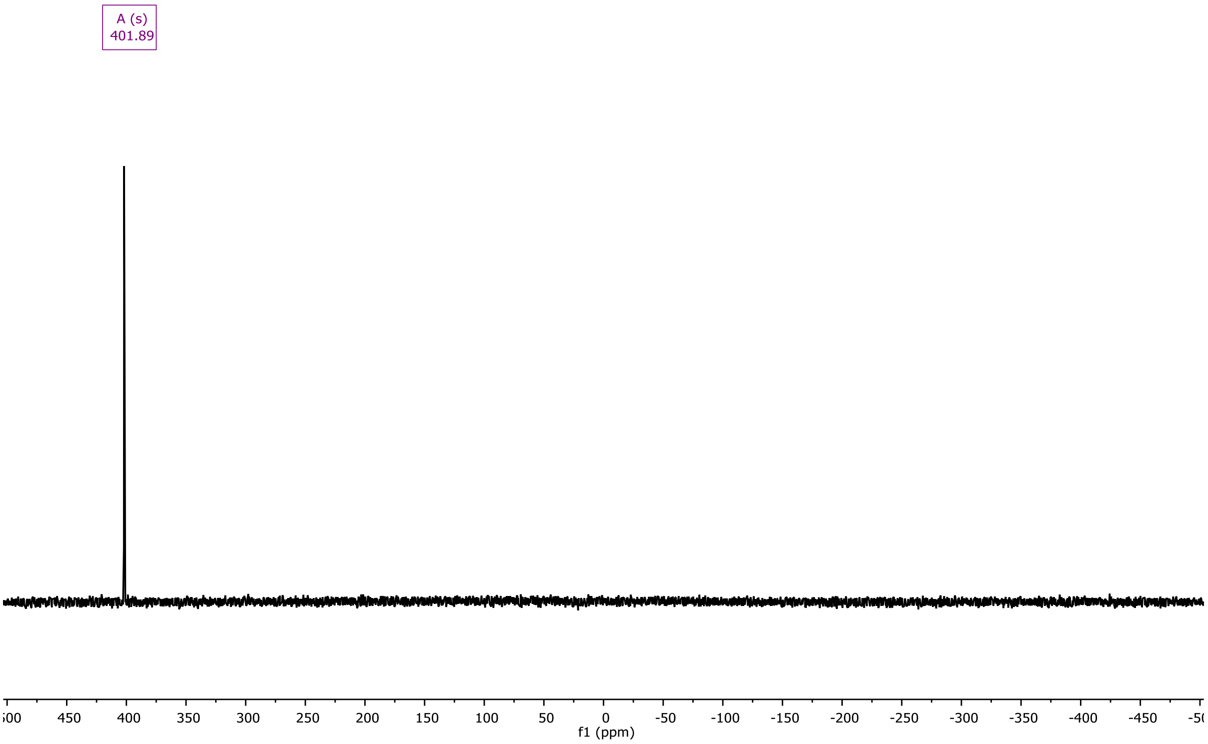

Figure S9. Room temperature ^{31}P NMR spectrum of **2** in C_6D_6.

1.3. NMR data for ([B]O){(C_6F_5)$_2$B(PMe_3)}C=P(C_6F_5) (**3**)

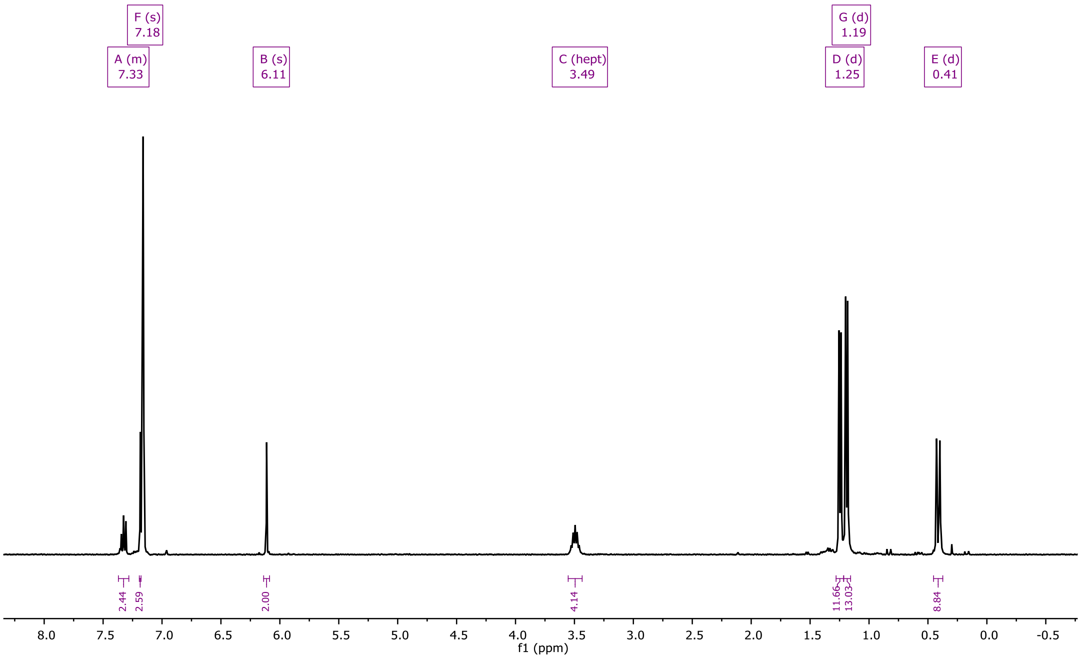

Figure S10. Room temperature ^{1}H NMR spectrum of **3** in C_6D_6.

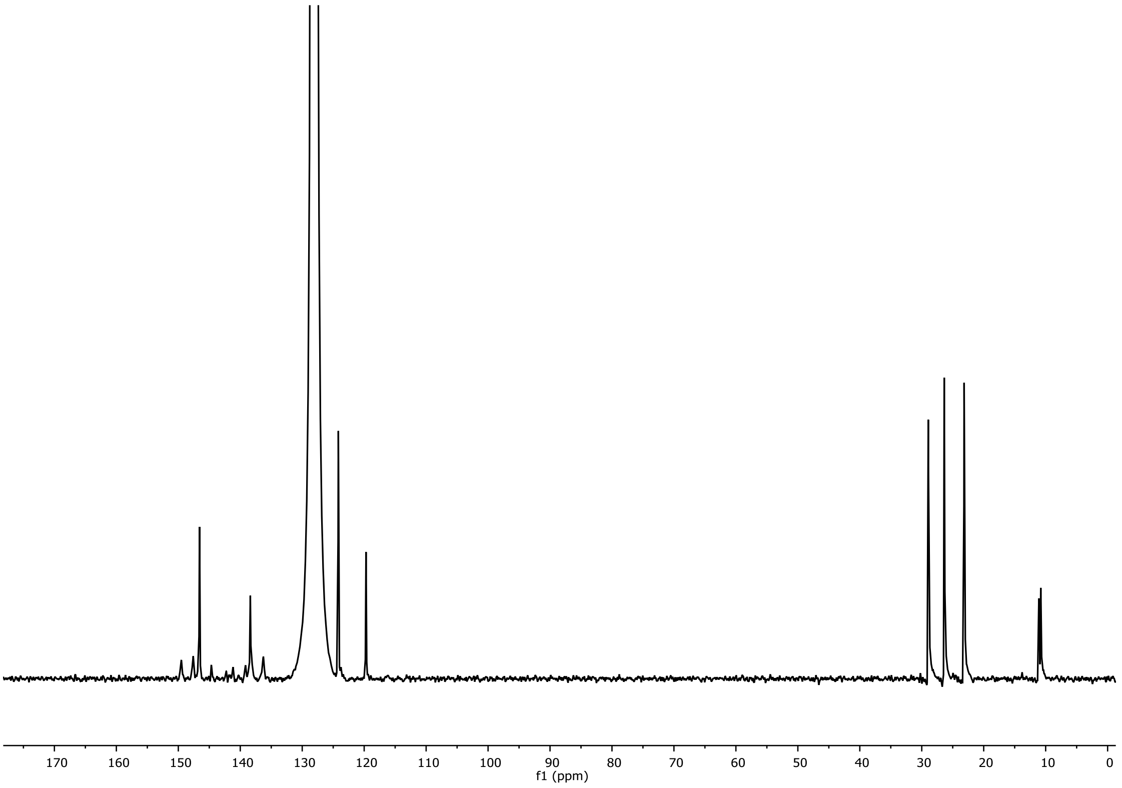

Figure S11. Room temperature ^{13}C{^{1}H} NMR spectrum of **3** in C_6D_6.

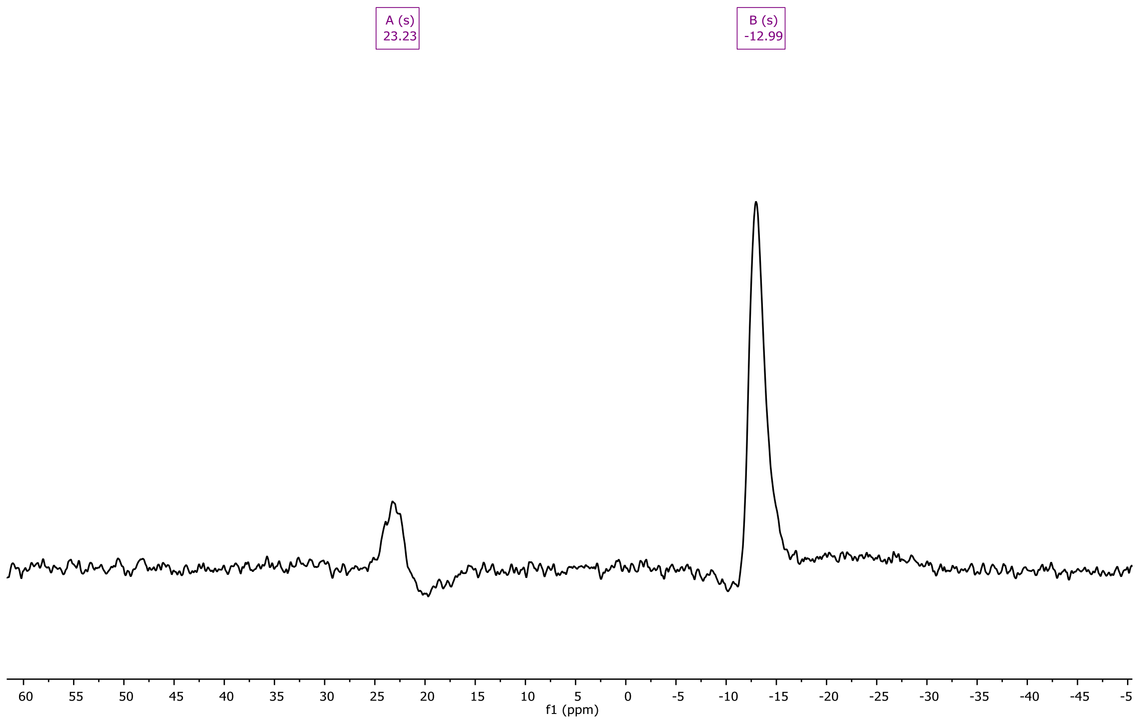

Figure S12. Room temperature ^{11}B NMR spectrum of **3** in C_6D_6.

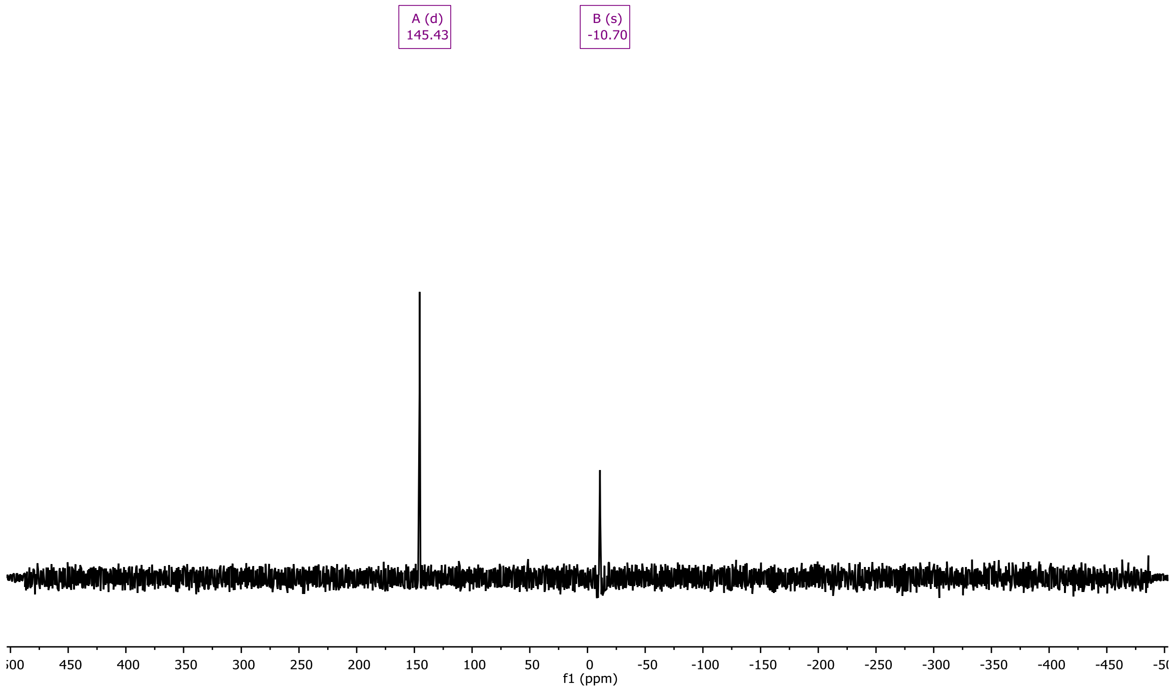

Figure S13. Room temperature ^{31}P NMR spectrum of **3** in C_6D_6.

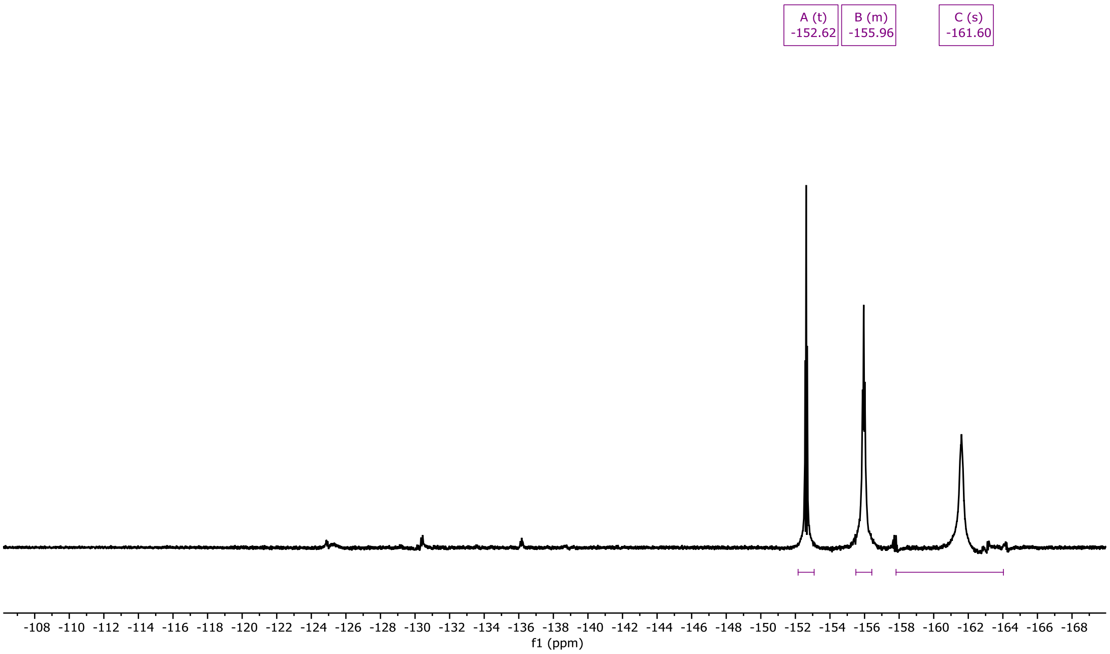

Figure S13. Room temperature ^{19}F NMR spectrum of **3** in C_6D_6.

1.4 NMR data for $(C_6F_5)P{=}C[B]\{OB(C_6F_5)_2\}$ (**4**)

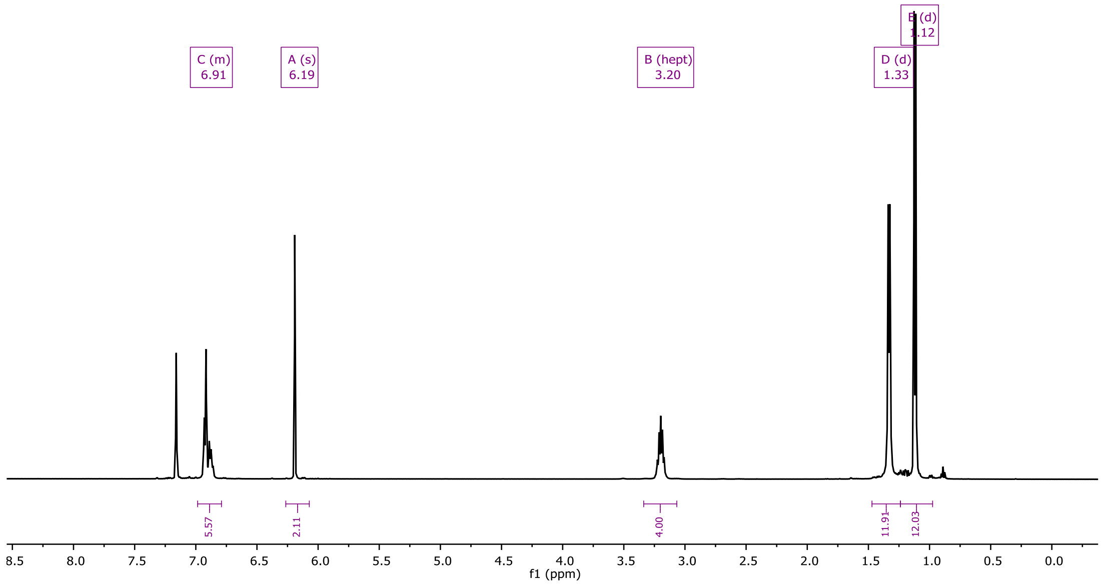

Figure S14. Room temperature ^{1}H NMR spectrum of **4** in C_6D_6.

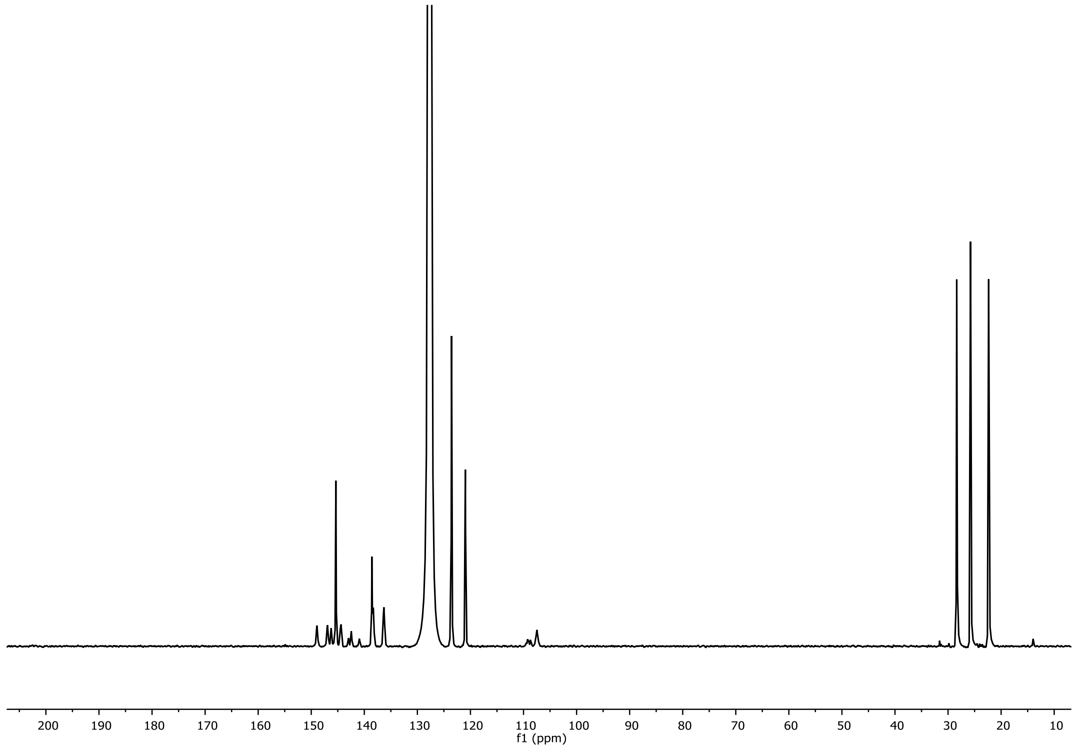

Figure S15. Room temperature $^{13}C\{^{1}H\}$ NMR spectrum of **4** in C_6D_6.

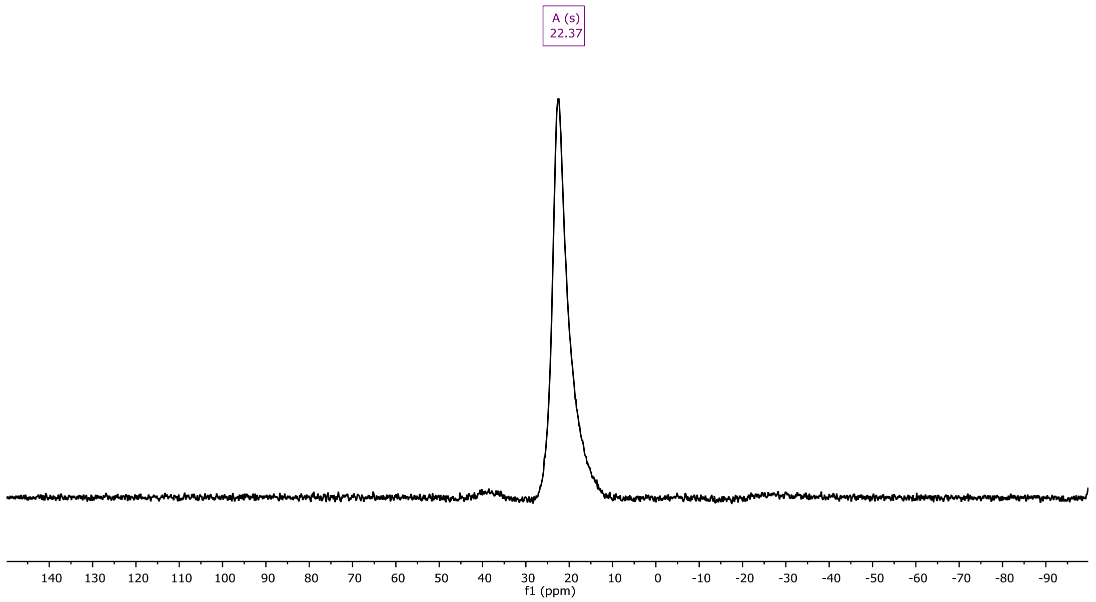

Figure S16. Room temperature ^{11}B NMR spectrum of **4** in C_6D_6.

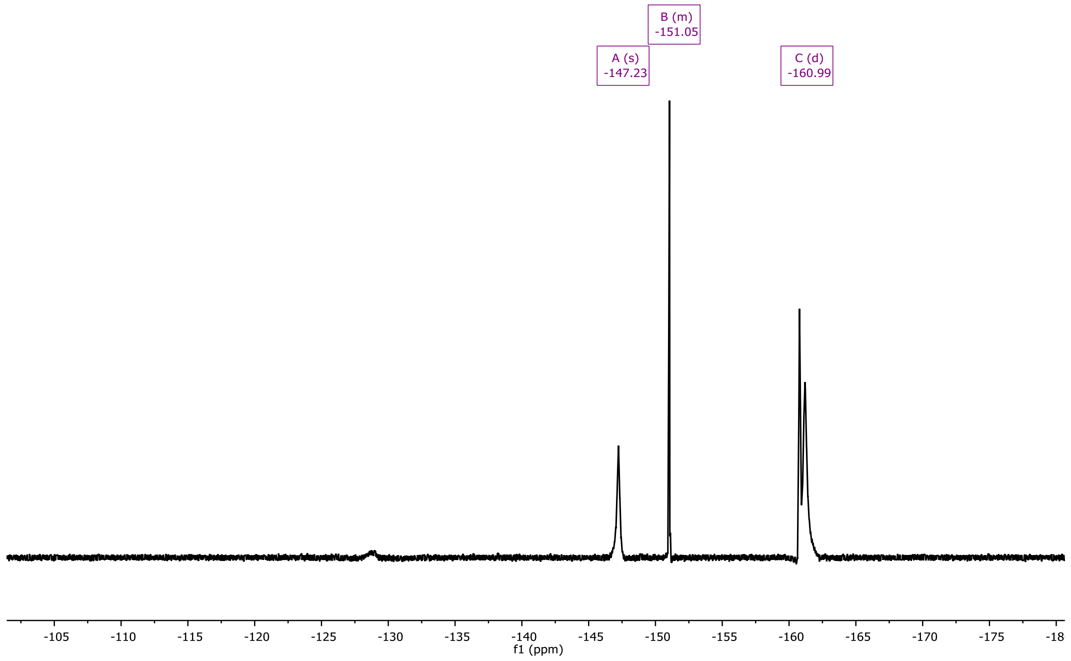

Figure S17. Room temperature ^{19}F NMR spectrum of **4** in C_6D_6.

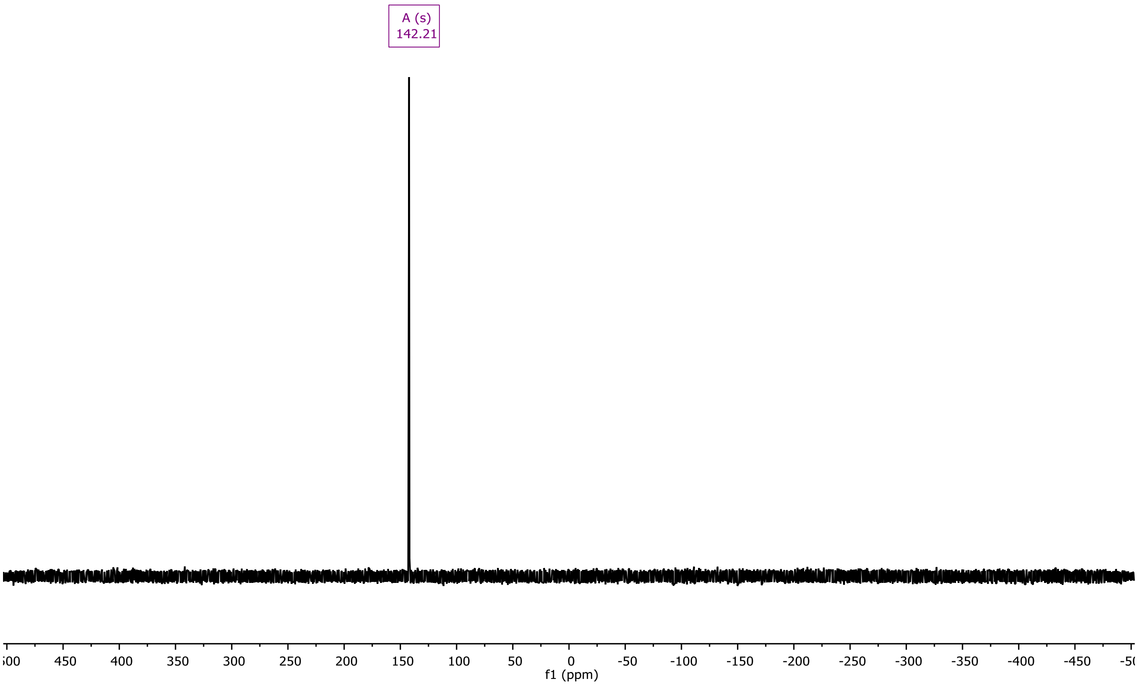

Figure S18. Room temperature ^{31}P NMR spectrum of **4** in C_6D_6.

2. Single crystal X-ray diffraction data

Table S1. Selected X-ray data collection and refinement parameters for **1**, **2** and **2**·0.5tol.

	**1**	**2**	**2**·0.5tol
Formula	$C_{45}H_{36}B_2F_{15}N_2OP$	$C_{45}H_{36}B_2F_{15}N_2OP$	$C_{48.5}H_{40}B_2F_{15}N_2OP$
CCDC	2001083	2001084	2001085
Fw [g mol^{-1}]	958.35	958.35	1004.41
Crystal system	monoclinic	triclinic	triclinic
Space group	*P*21/*n*	*P*–1	*P*–1
a (Å)	11.1556(2)	11.2406(2)	11.9988(6)
b (Å)	34.4444(4)	12.8123(4)	12.8341(5)
c (Å)	11.3887(1)	16.0763(3)	16.9819(4)
α (°)	90	95.774(2)	100.339(3)
β (°)	91.954(1)	95.372(2)	108.287(4)
γ (°)	90	105.189(2)	101.708(4)
V (Å^3)	4373.54(10)	2205.63(9)	2346.76(17)
Z	4	2	2
Radiation, λ (Å)	Cu Kα, 1.54184	Cu Kα, 1.54184	Cu Kα, 1.54184
Temp (K)	150(2)	150(2)	150(2)
ρ_{calc} (g cm^{-3})	1.455	1.443	1.421
μ (mm^{-1})	1.477	1.465	1.404
Reflections collected	36336	27341	28169
Independent reflections	9069	9098	9642
Parameters	603	603	642
R(int)	0.0374	0.0237	0.0376
R1/wR2,[a] I ≥ 2σI (%)	4.69/12.03	3.97/9.95	5.36/14.67
R1/wR2,[a] all data (%)	5.89/12.88	4.87/10.53	6.26/15.68
GOF	1.044	1.029	1.016

$R1 = [\Sigma||F_o| - |F_c||]/\Sigma|F_o|$; $wR2 = \{[\Sigma w[(F_o)^2 - (F_c)^2]^2]/[\Sigma w(F_o^2)^2\}^{1/2}$; $w = [\sigma^2(F_o)^2 + (AP)^2 + BP]^{-1}$, where $P = [(F_o)^2 + 2(F_c)^2]/3$ and the A and B values are 0.0616 and 1.73 for **1**, 0.0455 and 0.99 for **2**, and 0.0881 and 1.33 for **2**·0.5tol.

Table S2. Selected X-ray data collection and refinement parameters for **3** and **4**.

	**3**	**4**
Formula	$C_{48}H_{45}B_2F_{15}N_2OP_2$	$C_{45}H_{36}B_2F_{15}N_2OP$
CCDC	2001086	2001087
Fw [g mol^{-1}]	1034.42	958.35
Crystal system	triclinic	triclinic
Space group	*P*–1	*P*–1
a (Å)	10.0611(3)	13.6128(4)
b (Å)	12.6527(4)	17.7940(4)
c (Å)	19.1586(9)	19.2912(5)
α (°)	81.253(3)	104.027(2)
β (°)	75.996(4)	102.959(2)
γ (°)	88.193(3)	90.090(2)
V (Å^3)	2338.85(16)	4410.3(2)
Z	2	4
Radiation, λ (Å)	Cu Kα, 1.54184	Cu Kα, 1.54184
Temp (K)	150(2)	150(2)
ρ_{calc} (g cm^{-3})	1.469	1.443
μ (mm^{-1})	1.737	1.465
Reflections collected	28673	49435
Independent reflections	9686	18221
Parameters	642	1205
R(int)	0.0308	0.0206
R1/wR2,[a] I ≥ 2σI (%)	4.24/10.99	3.62/9.56
R1/wR2,[a] all data (%)	5.24/12.02	4.06/9.98
GOF	1.023	1.016

$R1 = [\Sigma||F_o| - |F_c||]/\Sigma|F_o|$; $wR2 = \{[\Sigma w[(F_o)^2 - (F_c)^2]^2]/[\Sigma w(F_o{}^2)^2]\}^{1/2}$; $w = [\sigma^2(F_o)^2 + (AP)^2 + BP]^{-1}$, where $P = [(F_o)^2 + 2(F_c)^2]/3$ and the A and B values are 0.0690 and 0.58 for **3** and 0.0523 and 1.30 for **4**.

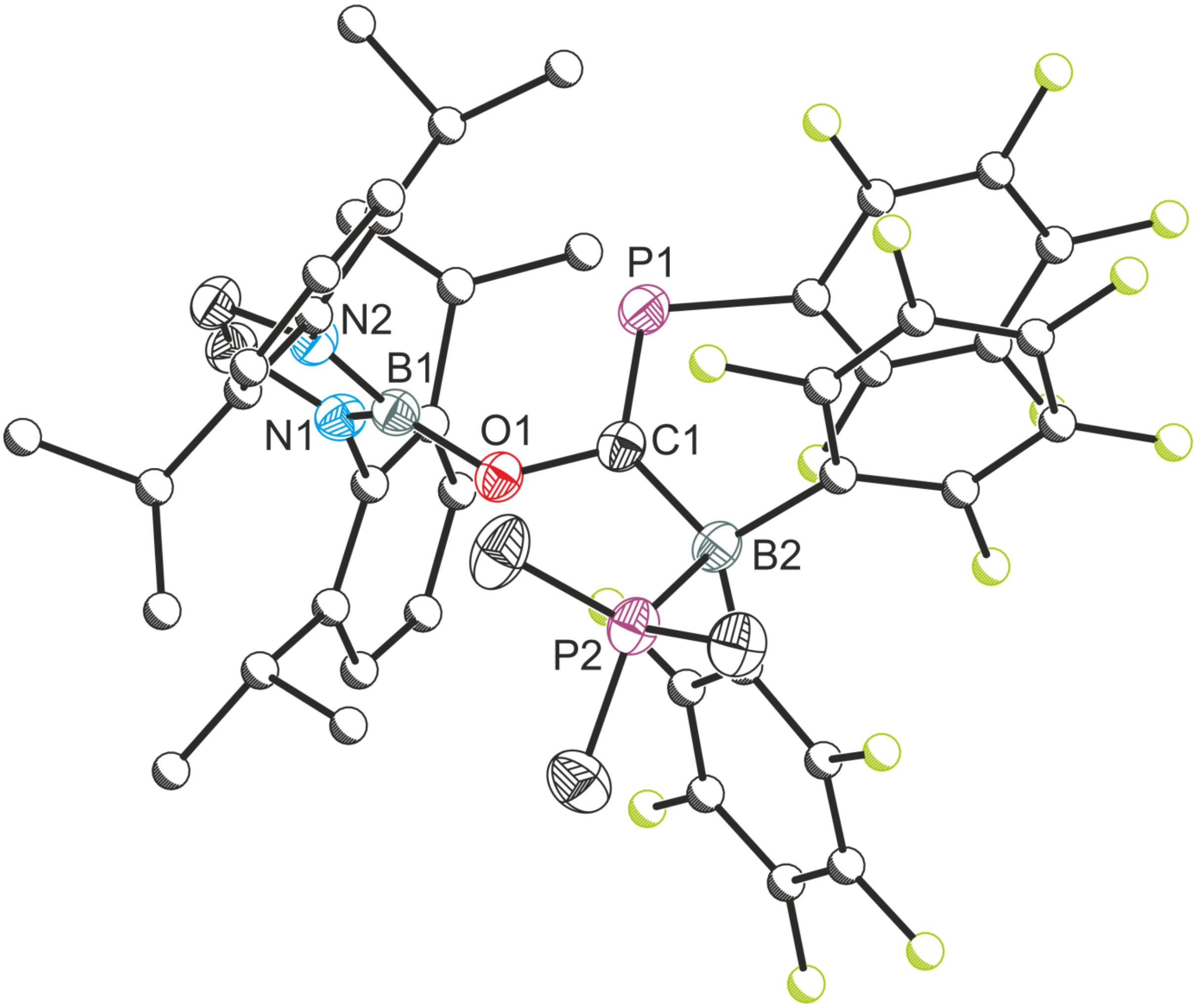

Figure S19. Molecular structure of **3**. Anisotropic displacement ellipsoids set at 50% probability. Hydrogen atoms have been omitted for clarity. Atoms of the Dipp and C_6F_5 moieties are pictured as spheres of arbitrary radius. Selected interatomic distances [Å] and angles [°]: B1–O1 1.411(2); O1–C1 1.378(2), C1–P1 1.7063(17), C1–B2 1.638(2), B2–P2: 2.0621(19); B1-O1-C1 126.97(13); O1-C1-P1 114.26(11), O1-C1-B2 113.43(13), P1-C1-B2 132.28(12).

3. Computational details

All calculations were done in G09.D1 version.[1] Optimizations were done using M06L/6-31G(d,p) in the gas phase.[2,3] The identities of minima and transition states were confirmed by the absence of imaginary frequencies or the presence of only one imaginary frequency, respectively. Transition states were connected to local minima through intrinsic reaction coordinate calculations. Natural bond order analysis was performed using NBO version 3.1.[4]

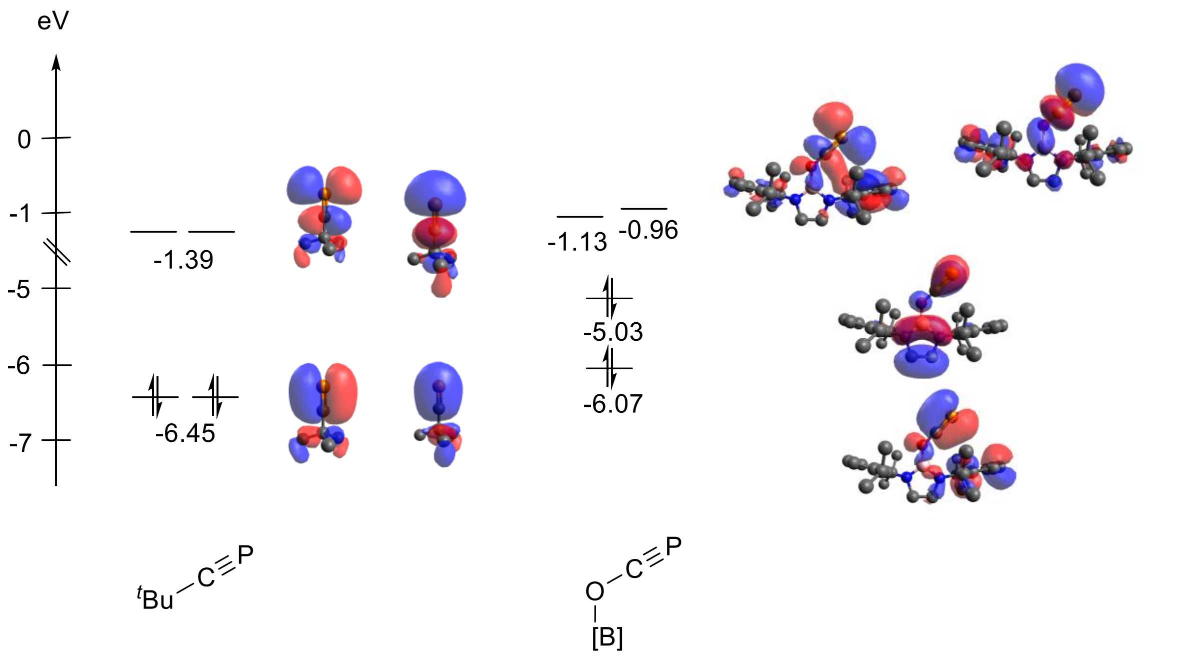

Figure S20. Frontier molecular orbital diagrams of ^{t}BuCP and [B]OCP from NBO analysis.

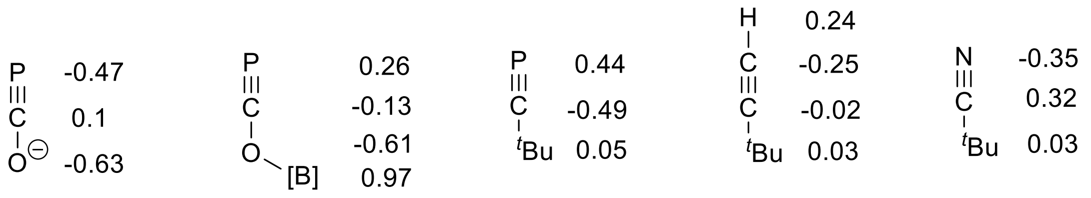

Figure S19. Natural population analysis charges (q) for [B]OCP, ^{t}BuCP and related species.

Geometry Optimised Cartesian Coordinates:

^{t}BuCP

Energy = -537.222609314
ZPE = -537.094918
Free Energy = -537.126239

C	0.741096	0.000221	-0.000003
P	2.293692	0.000117	-0.000040
C	-0.725435	0.000024	-0.000018
C	-1.232921	-0.430375	1.384909
C	-1.232926	-0.984379	-1.065004
H	-0.885932	-0.700637	-2.062109
H	-0.886081	-2.000781	-0.860602
H	-2.328227	-0.990163	-1.071370
H	-0.886255	-1.435929	1.637557
H	-2.328214	-0.432573	1.393225
H	-0.885669	0.254667	2.162896
C	-1.233529	1.414362	-0.319806
H	-0.886166	1.746228	-1.301816
H	-2.328833	1.422193	-0.322573
H	-0.887708	2.136117	0.424930

^{t}BuCN

Energy = -250.669602195
ZPE = -250.539035
Free Energy = -250.569266

C	-1.655723	-0.435646	0.070181
N	-2.695567	0.089954	0.119915
C	-0.344141	-1.097848	0.007585
C	-0.271272	-1.927931	-1.280054
C	-0.191564	-2.008105	1.232505
H	-0.248244	-1.437294	2.162809
H	-0.967645	-2.777270	1.256369
H	0.782310	-2.505987	1.195581
H	-1.048911	-2.695514	-1.303764
H	0.701991	-2.425152	-1.336594
H	-0.385481	-1.298972	-2.166548
C	0.751405	-0.024894	0.007053
H	0.713225	0.584044	0.913837
H	1.732173	-0.508224	-0.039317
H	0.657265	0.640374	-0.854961

^{t}BuCCH

Energy = -234.566097706
ZPE = -234.425402
Free Energy = -234.455789

C	-1.673457	-0.469697	0.041279
C	-2.763974	0.054256	0.079184
H	-3.723459	0.514033	0.111197
C	-0.353846	-1.107131	-0.002088
C	-0.236797	-1.948524	-1.280574
C	-0.185092	-2.012916	1.225264

H	-0.262659	-1.439527	2.153073
H	-0.948387	-2.795587	1.246249
H	0.798398	-2.494483	1.201804
H	-1.001135	-2.729830	-1.310320
H	0.746415	-2.429412	-1.322870
H	-0.352268	-1.327959	-2.173479
C	0.735492	-0.026506	0.003164
H	0.680090	0.586288	0.907139
H	1.725445	-0.493886	-0.032822
H	0.638987	0.636286	-0.861150

$OCP^{–}$

Energy = -454.757627995
ZPE = -454.750208
Free Energy = -454.771616

O	-0.666422	-1.026711	-0.001085
C	-1.701676	-0.424759	-0.000274
P	-3.119310	0.351547	0.000465

BCF

Energy = -2208.03953551
ZPE = -2207.884959
Free Energy = -2207.946751

B	0.002572	-0.003417	0.002956
C	1.551670	-0.202902	0.000571
C	-0.948116	-1.243109	0.007621
C	-0.595110	1.440524	-0.001441
C	2.420187	0.663418	-0.678315
C	2.175690	-1.260893	0.676719
C	-0.635293	-2.435454	-0.661594
C	-2.180704	-1.243252	0.676216
C	-1.772673	1.765904	-0.689077
C	0.005899	2.505619	0.684239
F	1.933616	1.698739	-1.374882
C	3.797123	0.494942	-0.703172
F	1.444166	-2.139732	1.374199
C	3.550254	-1.449157	0.694699
F	0.509291	-2.545328	-1.347654
C	-1.476326	-3.539196	-0.685316
F	-2.575104	-0.165929	1.367376
C	-3.038070	-2.334107	0.693147
F	-2.420536	0.831286	-1.396667
C	-2.315144	3.042544	-0.710491
F	1.126578	2.308334	1.390995
C	-0.519671	3.789255	0.707872
F	4.577476	1.335681	-1.381201
C	4.365146	-0.566689	-0.006419
F	4.093777	-2.461456	1.369473
F	-1.138598	-4.643259	-1.350354
C	-2.684926	-3.487161	0.000234
F	-4.193306	-2.287427	1.355084
F	-3.427819	3.300544	-1.396921
C	-1.687132	4.059422	0.001349
F	0.079001	4.760412	1.396113

F	5.681352	-0.737722	-0.012850
F	-3.498781	-4.535331	-0.006196
F	-2.201078	5.283283	0.007280

[B]OCP

Energy = -667.661013401
ZPE = -667.578583
Free Energy = -667.611226

O	0.634477	-1.063052	0.002327
N	-1.874129	-1.049468	-0.001645
N	-0.881235	0.988889	0.002036
C	-2.268249	1.156441	0.000149
C	-2.860983	-0.060428	-0.002184
B	-0.612826	-0.401982	0.001078
H	-2.726482	2.133308	0.000109
H	-3.912310	-0.302039	-0.004129
H	-2.103810	-2.026402	-0.003167
H	-0.247527	1.768343	0.005428
C	1.740503	-0.407800	0.004201
P	3.106558	0.349060	-0.002533

TS1

Energy = -2875.67203233
ZPE = -2875.434833
Free Energy = -2875.503135

O	-1.956869	1.422050	-0.982463
N	-3.855064	2.887781	-1.679738
N	-3.543207	1.037151	-2.953250
C	-4.644933	1.749230	-3.438808
C	-4.824235	2.857459	-2.681725
B	-3.031875	1.744862	-1.831539
H	-5.213628	1.410982	-4.290708
H	-5.565925	3.633669	-2.786491
H	-3.794476	3.642617	-1.020551
H	-3.312069	0.131852	-3.322469
C	-1.040071	0.563219	-1.345372
P	-0.370050	-0.375783	-2.496841
B	-0.005255	-0.053764	-0.167553
C	1.245056	-0.983119	-0.843896
C	-1.128898	-0.843395	0.705332
C	0.788982	1.153096	0.561279
C	2.315699	-0.360345	-1.534400
C	1.563987	-2.273422	-0.368163
C	-1.771265	-2.004291	0.256138
C	-1.706043	-0.288995	1.854119
C	0.932672	2.457884	0.101983
C	1.507510	0.846463	1.717327
F	2.180286	0.895138	-1.981790
C	3.519686	-0.976505	-1.832276
F	0.759732	-2.935723	0.462378
C	2.766278	-2.914576	-0.641485
F	-1.363041	-2.621772	-0.870123
C	-2.845804	-2.603667	0.898852
F	-1.250697	0.862481	2.372462

C	-2.778715	-0.861273	2.533031
F	0.341013	2.857121	-1.038340
C	1.712683	3.409248	0.753940
F	1.445897	-0.403681	2.214996
C	2.287290	1.764883	2.404587
F	4.458016	-0.336557	-2.529505
C	3.748842	-2.271779	-1.383367
F	3.006041	-4.127456	-0.146943
F	-3.393533	-3.719847	0.409895
C	-3.357815	-2.027092	2.052823
F	-3.264935	-0.284169	3.634793
F	1.822247	4.647562	0.264141
C	2.392878	3.062087	1.912750
F	2.933462	1.417871	3.520515
F	4.898481	-2.879066	-1.645346
F	-4.392207	-2.579109	2.684849
F	3.148419	3.960602	2.543899

1DFT

Energy = -2875.72915034
ZPE = -2875.489818
Free Energy = -2875.559428

C	1.093086	1.438691	-0.269526
C	-0.960097	1.363515	1.470364
P	0.405251	2.475875	0.919938
F	2.974766	0.029067	1.053575
O	2.215885	1.789179	-0.931085
F	-2.602874	2.725849	0.440721
F	0.533689	-0.164538	2.506238
F	4.390205	-4.265084	-0.094457
F	-0.766171	1.783249	-2.444764
F	-1.192818	-2.180095	0.119479
F	4.736331	-1.945065	1.283570
F	-4.593440	1.087188	1.210088
F	0.468418	-2.607789	-2.009225
F	-4.043860	-1.160592	2.635698
F	2.252744	-4.565577	-1.746873
F	-3.374279	1.645459	-3.066026
N	2.773006	4.280501	-0.946977
F	-1.468713	-1.769675	3.282395
N	4.176896	2.913465	0.182444
F	-3.808104	-2.263596	-0.467702
F	-4.909459	-0.365153	-2.075872
C	3.507015	-3.282991	-0.219124
C	2.749822	-1.077452	0.336993
C	1.614474	-1.181129	-0.486346
C	4.650258	4.221529	0.270733
C	3.679604	-2.096111	0.484872
C	3.809525	5.040659	-0.404820
B	0.604322	-0.010542	-0.640627
C	-0.862526	-0.195912	-1.139588
C	2.408163	-3.435687	-1.058669
C	-3.336681	0.821725	1.571417
C	-2.295423	1.654413	1.184580
C	-1.481508	0.766900	-1.946690
B	2.977397	2.921349	-0.580689
C	-3.044734	-1.298796	-0.980845

C	-1.689640	-1.231982	-0.690578
C	-0.722887	0.203539	2.211032
C	-1.743536	-0.646370	2.621236
C	1.489994	-2.401475	-1.172477
C	-2.827800	0.716131	-2.284458
C	-3.057482	-0.337960	2.291077
C	-3.615402	-0.316223	-1.785467
H	5.551950	4.471730	0.808094
H	3.872269	6.108667	-0.544917
H	2.023726	4.704203	-1.464165
H	4.635011	2.149147	0.645029

TS2

Energy = -2875.64990887
ZPE = -2875.413018
Free Energy = -2875.479566

O	1.424739	-0.273640	-2.695781
N	3.866332	-0.494375	-2.104724
N	2.932541	1.520431	-1.632247
C	4.261504	1.520794	-1.208321
C	4.819351	0.318256	-1.492696
B	2.658624	0.239282	-2.177869
H	4.714164	2.385047	-0.747791
H	5.827326	-0.021786	-1.312160
H	4.044214	-1.461914	-2.308969
H	2.310571	2.278324	-1.406341
C	0.065816	0.288429	-1.782545
P	0.223791	0.970990	-3.267530
B	-0.368778	-0.032396	-0.284806
C	-1.351780	1.283262	-0.149496
C	0.942947	-0.066864	0.688172
C	-1.124862	-1.460493	-0.138522
C	-2.749172	1.241541	-0.090920
C	-0.816700	2.576181	-0.150212
C	1.925148	-1.029199	0.446483
C	1.199324	0.761591	1.778658
C	-1.174225	-2.455102	-1.106214
C	-1.706442	-1.782894	1.085363
F	-3.416457	0.081098	-0.143551
C	-3.541200	2.381449	0.027768
F	0.522128	2.749983	-0.251392
C	-1.565958	3.737094	-0.037197
F	1.748173	-1.930168	-0.545745
C	3.119972	-1.113877	1.147160
F	0.305738	1.682804	2.173398
C	2.376300	0.700774	2.521504
F	-0.621965	-2.255103	-2.318815
C	-1.768326	-3.695560	-0.891852
F	-1.706793	-0.865963	2.070726
C	-2.316305	-3.002424	1.342276
F	-4.868778	2.280316	0.103663
C	-2.948738	3.636261	0.056048
F	-0.980640	4.937234	-0.032279
F	4.043943	-2.022066	0.813112
C	3.350309	-0.234112	2.196342
F	2.576806	1.536244	3.544530
F	-1.782297	-4.623312	-1.854582

C	-2.344636	-3.968449	0.341303
F	-2.871579	-3.261593	2.529159
F	-3.697269	4.730357	0.163853
F	4.491560	-0.289848	2.882539
F	-2.921053	-5.149110	0.568499

I2$_{DFT}$

Energy = -2875.76439136
ZPE = -2875.525676
Free Energy = -2875.594453

O	-1.230948	-0.260775	-2.209905
N	-3.729199	-0.031662	-2.240329
N	-2.940610	-2.099919	-1.762014
C	-4.335401	-2.116141	-1.691726
C	-4.804456	-0.876891	-1.975665
B	-2.515534	-0.776123	-2.110082
H	-4.889750	-3.011568	-1.455337
H	-5.826896	-0.532494	-2.001982
H	-3.859666	0.946518	-2.425565
H	-2.401730	-2.927757	-1.576780
C	0.854731	-0.752686	-0.851814
P	0.126221	-1.269882	-2.291123
B	-0.018929	0.283879	-0.050443
C	2.144828	-1.183770	-0.358505
C	-1.276039	-0.260033	0.705986
C	0.331662	1.809532	-0.068440
C	2.578099	-0.821905	0.928797
C	3.025087	-2.006517	-1.079899
C	-2.497988	0.423030	0.670593
C	-1.326936	-1.545291	1.255072
C	0.896863	2.465632	-1.168522
C	0.130695	2.605707	1.067016
F	1.776679	-0.063450	1.698134
C	3.791011	-1.226760	1.462218
F	2.674852	-2.415176	-2.305157
C	4.248167	-2.421588	-0.578315
F	-2.569373	1.649057	0.129406
C	-3.693059	-0.133723	1.101395
F	-0.209976	-2.283585	1.348935
C	-2.497401	-2.122532	1.728960
F	1.131902	1.810425	-2.311348
C	1.215407	3.819245	-1.159148
F	-0.372979	2.063602	2.183439
C	0.458218	3.952488	1.120649
F	4.149553	-0.861888	2.692423
C	4.634011	-2.032008	0.701614
F	5.051406	-3.198024	-1.302897
F	-4.838111	0.538570	0.983335
C	-3.692571	-1.417926	1.630869
F	-2.490416	-3.348330	2.258713
F	1.728190	4.407434	-2.240972
C	0.999326	4.563336	-0.005838
F	0.263893	4.665291	2.230896
F	5.798379	-2.435497	1.198799
F	-4.827386	-1.970841	2.047031
F	1.309806	5.855171	0.024039

2DFT

Energy = -2875.75984059
ZPE = -2875.520734
Free Energy = -2875.592998

P	-1.409413	1.839143	-0.314644
F	-2.865398	-0.713396	-1.588649
F	-0.181697	-1.263597	2.268213
F	0.044789	-1.706919	-2.063937
F	3.057448	-0.680405	1.440203
O	-3.022142	1.616337	-0.143398
F	-4.186821	-2.975821	-0.987342
F	2.827153	0.953331	-1.950049
F	0.699198	2.333406	2.052667
F	-1.506032	-3.548415	2.850366
F	4.439418	3.104367	-1.821125
F	4.188671	4.870681	0.233232
F	-3.518428	-4.405985	1.227748
F	2.314423	4.474944	2.155309
F	4.287071	-3.048468	1.061878
F	1.280041	-4.066662	-2.425072
F	3.399979	-4.751975	-0.862765
N	-5.389295	2.360283	-0.417844
N	-3.846793	4.016256	-0.479729
C	-0.742241	0.330121	0.044638
C	-1.507693	-0.883205	0.345480
C	1.675679	1.566241	0.026500
C	-2.541322	-1.360394	-0.466279
C	-1.175462	-1.661971	1.460467
C	1.498349	-1.102235	-0.289059
C	1.585570	2.503088	1.061442
C	1.075404	-2.012905	-1.259875
C	-3.218346	-2.538642	-0.180205
C	-5.118482	4.569772	-0.637788
H	-5.270392	5.631031	-0.759961
C	-6.040188	3.577975	-0.602155
H	-7.113051	3.648863	-0.691634
C	3.517010	2.904583	-0.880051
C	2.675390	1.802252	-0.922827
C	2.604143	-1.489393	0.470751
C	-1.842363	-2.838716	1.772552
C	2.421928	3.609511	1.146611
C	3.388085	3.811460	0.167143
C	-2.872337	-3.277015	0.946339
C	3.247829	-2.707749	0.299900
C	1.702554	-3.233838	-1.472598
B	-3.988475	2.601362	-0.336712
C	2.792916	-3.582639	-0.681858
B	0.787927	0.275387	-0.055367
H	-5.892334	1.492332	-0.379627
H	-3.027410	4.596801	-0.469388

4. References

[1] M. J. Frisch, G. W. Trucks, H. B. Schlegel, G. E. Scuseria, M. A. Robb, J. R. Cheeseman, G. Scalmani, V. Barone, B. Mennucci, G. A. Petersson, H. Nakatsuji, M. Caricato, X. Li, H. P. Hratchian, A. F. Izmaylov, J. Bloino, G. Zheng, J. L. Sonnenberg, M. Hada, M. Ehara, K. Toyota, R. Fukuda, J. Hasegawa, M. Ishida, T. Nakajima, Y. Honda, O. Kitao, H. Nakai, T. Vreven, J. J. A. Montgomery, J. E. Peralta, F. Ogliaro, M. Bearpark, J. J. Heyd, E. Brothers, K. N. Kudin, V. N. Staroverov, R. Kobayashi, J. Normand, K. Raghavachari, A. Rendell, J. C. Burant, S. S. Iyengar, J. Tomasi, M. Cossi, N. Rega, J. M. Millam, M. Klene, J. E. Knox, J. B. Cross, V. Bakken, C. Adamo, J. Jaramillo, R. Gomperts, R. E. Stratmann, O. Yazyev, A. J. Austin, R. Cammi, C. Pomelli, J. W. Ochterski, R. L. Martin, K. Morokuma, V. G. Zakrzewski, G. A. Voth, P. Salvador, J. J. Dannenberg, S. Dapprich, A. D. Daniels, O. Farkas, J. B. Foresman, J. V. Ortiz, J. Cioslowski, D. J. Fox, *Gaussian 09, Revision D.01*. Gaussian, Inc., Wallingford CT, **2009**.

[2] Y. Zhao, D. G. Truhlar, *J. Chem. Phys.* **2006**, *125*,194101: 1–18.

[3] V. A. Rassolov, M. A. Ratner, J. A. Pople, P. C. Redfern, L. A. Curtiss, *J. Comp. Chem.* **2001**, *22*, 976–84.

[4] (a) A. E. Reed, L. A. Curtiss, F. Weinhold, *Chem. Rev.* **1988**, *88*, 899–926; (b) E. D. Glendening, J. K. Badenhoop, A. E. Reed, J. E. Carpenter, J. A. Bohmann, C. M. Morales, F. Weinhold, *NBO 6.0*, Theoretical Chemistry Institute, University of Wisconsin, Madison, WI, **2009**.
